# A Digital Serious Game (Anticip’action) to Support Advance Care Planning Discussions in the General Population: Usability Study

**DOI:** 10.2196/73378

**Published:** 2025-08-21

**Authors:** Dafne Campioni, Frederic Ehrler, Antoine Berger, Christine Clavien

**Affiliations:** 1 Institute for Ethics, History, and the Humanities Faculty of Medicine University of Geneva Geneva Switzerland; 2 Department of Medical Information Sciences University Hospital of Geneva Geneva Switzerland

**Keywords:** advance care planning, mobile health, mHealth, mobile apps, mobile phone, palliative care, usability, serious game, conversation tool, end of life

## Abstract

**Background:**

In the context of an aging population and increasingly medicalized end-of-life practices, it is crucial to promote early discussions to help patients express their view on what is essential in their life as well as articulate their preferences regarding future medical treatments and end-of-life issues. An interprofessional research team at Geneva University and the Geneva University Hospitals has developed Anticip’action, a card game designed to help initiate and conduct advance care planning and end-of-life discussions. It is available for free in paper format in diverse languages and in a digital version in French.

**Objective:**

This study aims to assess the ergonomic quality of the digital version of the game with primary users.

**Methods:**

Overall, 10 users (women: n=5, 50%; men: n=5, 50%; mean age 41, SD 13.4 years; range 25-65 years; education: upper level; comfortable with smartphones) completed an online usability test. The test began with a rapid desirability test to capture initial impressions of the game’s main screen without knowing what it is about. This was followed by a think-aloud procedure, including 26 tasks to perform all steps of the game. Posttest questionnaires were administered to collect participants’ subjective perceptions of the usability (System Usability Scale), attractiveness (AttrakDiff), and relevance as well as overall endorsement of the game (Mobile Application Rating Scale). Open-ended questions were used to further explore usability issues. Usability problems were categorized and evaluated using standard evaluation grids. Content readability was assessed with Scolarius.

**Results:**

The rapid desirability test revealed an overall good or average impression. In 83.2% (208/250) of the cases, participants successfully completed the think-aloud tasks without assistance. Some of the tasks (4/25, 16%) revealed multiple usability issues requiring assistance. Analysis of the 23 failures and difficulties encountered revealed that 3 (13%) issues were due to suboptimal wording of the task instructions and that there were 9 (39%) major usability problems. All could be addressed through minor modifications. The Scolarius test indicated that the card titles were understandable at an elementary school level, while the explanations on the back of the cards required a high school reading level. Participants rated Anticip’action as good or excellent in usability (79 out of 100 on the System Usability Scale), attractiveness (1.57 on the −3 to +3 AttrakDiff scale), and relevance (4.1 out of 5 on section F of the Mobile Application Rating Scale). Participants provided overall positive qualitative feedback.

**Conclusions:**

The usability testing of the digital French version of Anticip’action produced positive results, with some areas for improvement identified. It can be recommended as a valuable resource for patients, families, and caregivers to prompt reflection, raise awareness, and support advance care planning conversations. Further tests should be conducted on wider population groups, including older patients and individuals less comfortable with digital solutions.

## Introduction

### Background

In the context of an aging population and increasingly medicalized end-of-life practices, it is crucial to promote early discussions to help patients express their view on what is essential in their life as well as articulate their preferences regarding future medical treatments and end-of-life issues [1,2]. Advance care planning and end-of-life discussions may include clarifying one’s own values and priorities in life; preferences regarding situations or medical treatments that are expected or not desired; and other important matters that should be addressed in case of loss of decision-making capacity or death, such as the management of financial matters, funeral planning, organ donation, the designation of a surrogate medical decision maker, and so on. These discussions are mostly relevant for patients facing a life-threatening illness or for older people with increased fragility. They help increase patient empowerment and decrease distress among loved ones and health professionals when important decisions have to be made [3]. However, these anticipatory discussions are often delayed or avoided due to a multitude of barriers such as health care professionals’ lack of trust in their communication abilities, time constraints, emotional burden, and fear of generating misunderstandings or conflicts [4-7]. To overcome these obstacles, it is crucial to develop strategies and communication tools that facilitate the initiation and conduction of end-of-life discussions.

Studies indicate that free online digital tools (websites, apps, online games, etc) could enhance interest, communication, and decision-making related to advance care planning [8-12]. These communication tools may serve as icebreakers, provide a supportive structure for conducting a discussion, and save clinicians’ time. Indeed, digital tools are easy to share with patients and can provide relevant information and input to conduct part of the reflection in a private and familial context ahead of the medical consultation. However, with some exceptions [13], existing solutions are mostly in English and not necessarily adapted to the European cultural context.

An interprofessional research team at Geneva University and the Geneva University Hospitals (HUG), composed of ethicists, physicians, nurses, trained patients as partners, and IT professionals, has developed information and communication tools to support the culture of advance care planning. In particular, the digital tool Accordons-nous (“Let’s agree”) is available for free as a module integrated into the larger Concerto application available on the HUG website [14]. The Concerto app can also be downloaded from the Google Play Store and Apple App Store. Accordons-nous [15] is currently only available in French. It includes information on advance care planning, discussion prompts, an advance care directive form, and a digital version of the card game Anticip’action*.*

### Anticip’action, the Card Game

**Figure 1:**
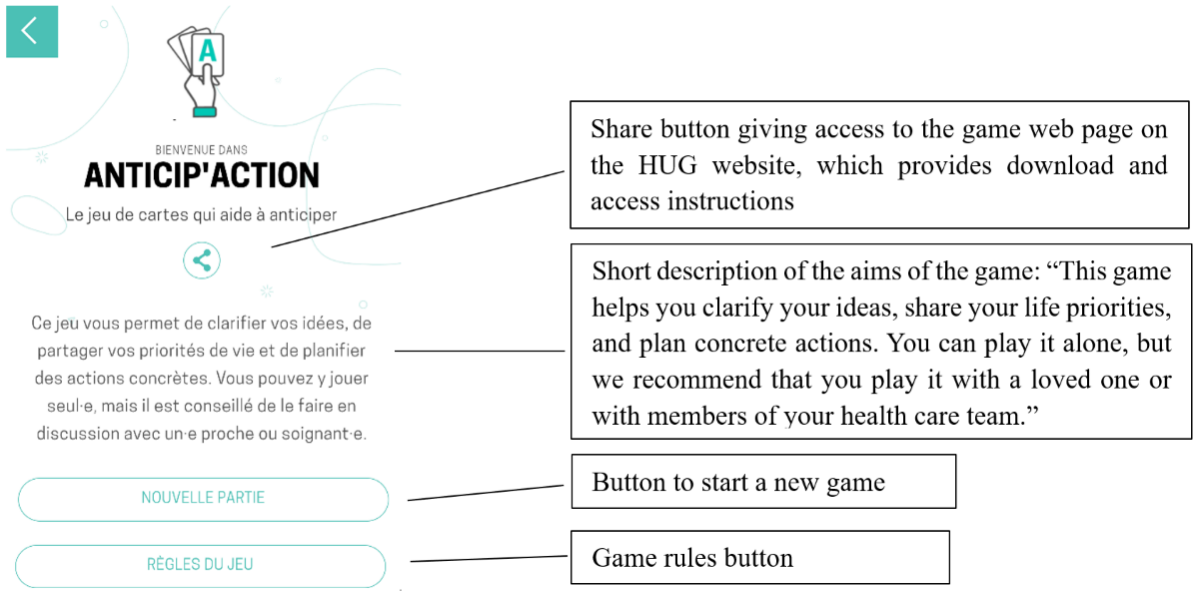
Welcome page of Anticip’action included in the Accordons-nous module. HUG: Geneva University Hospitals.

**Figure 2:**
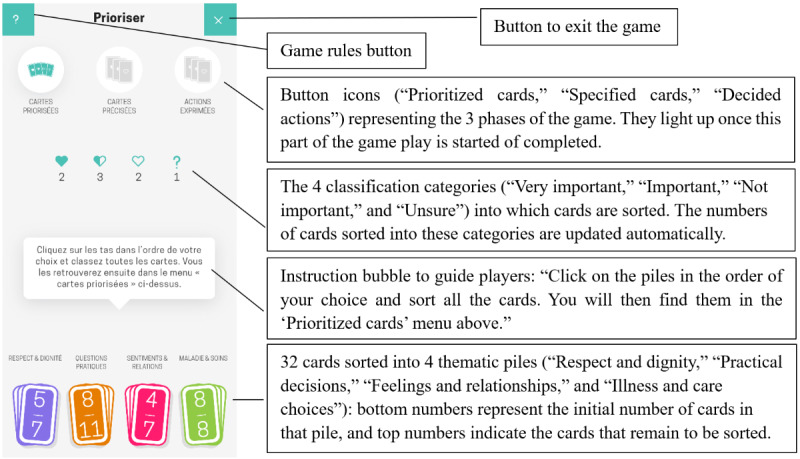
Main screen of the game.

**Figure 3:**
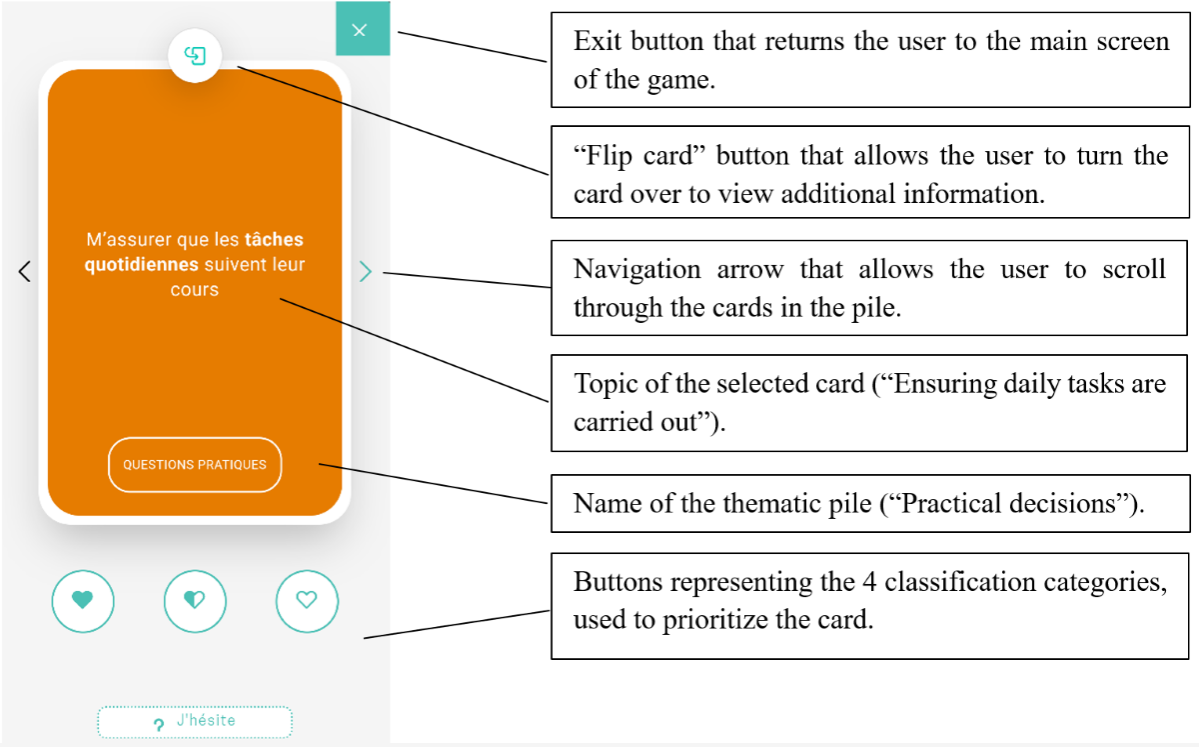
Game phase 1—“Prioritize”: screen for sorting cards allocated to the theme “Practical decisions.”.

**Figure 4:**
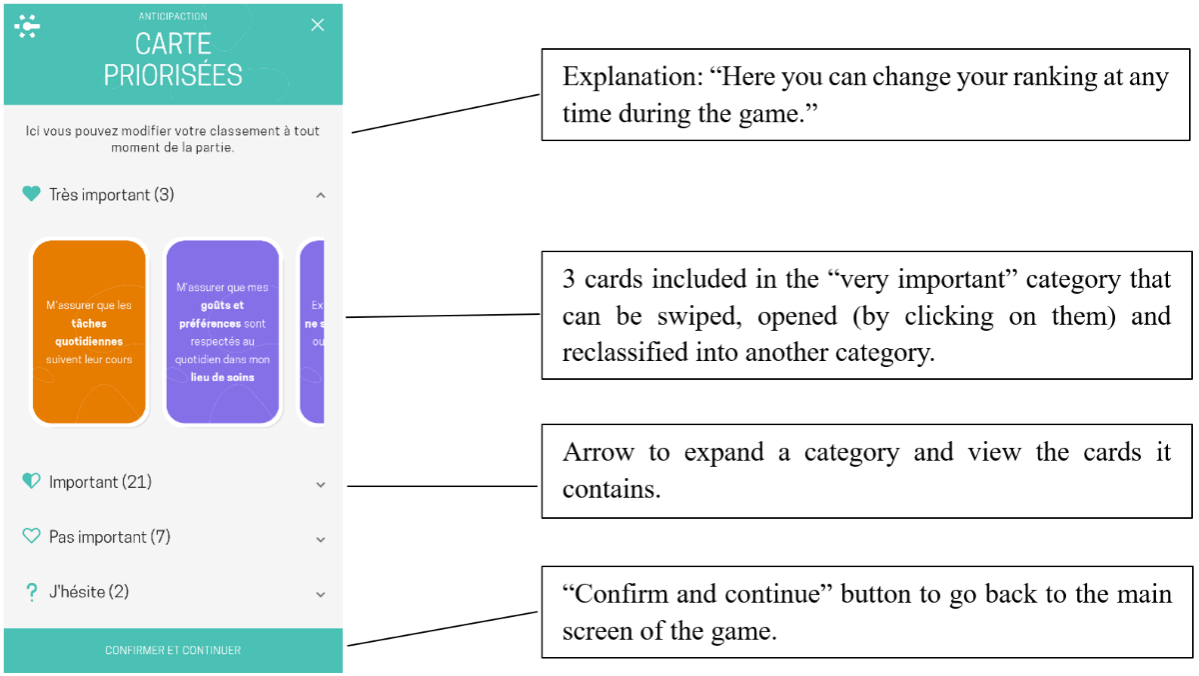
“Prioritized cards” screen, which allows users to re-sort cards into different categories at any time during the game. This screen can be accessed from the main screen by clicking on the “Prioritized cards” button icon.

**Figure 5:**
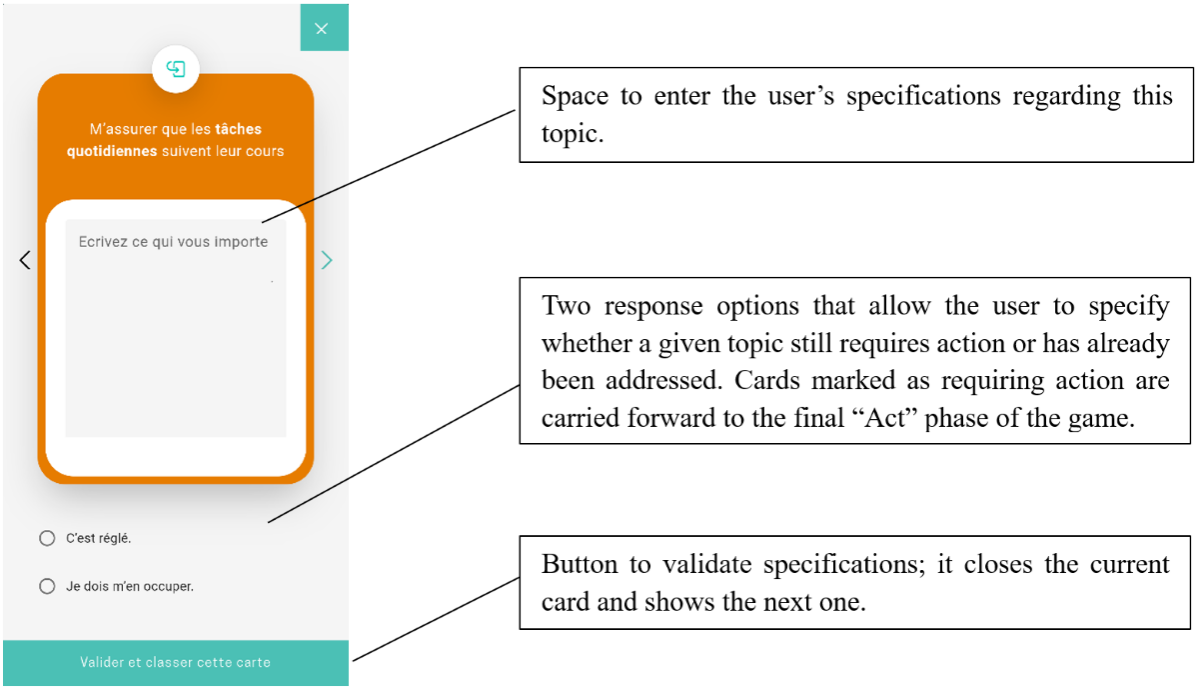
Game phase 2: “Specify”—screen that allows users to specify reasons for having marked a card as “Very important.”.

**Figure 6:**
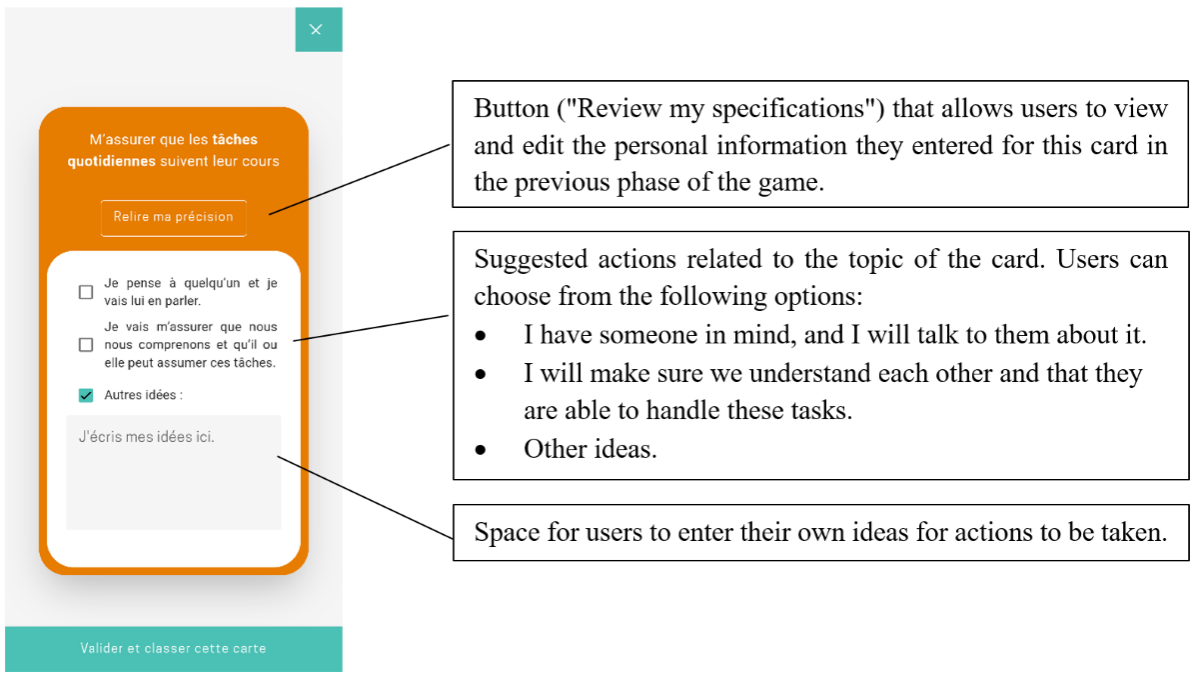
Game phase 3: “Act”—screen that allows users to choose actions to complete in relation to the topic of the card.

Anticip’action is a card game designed to help users clarify their values and priorities in life, discuss them with their loved ones and health care professionals, and plan concrete actions regarding care and end-of-life decisions. It is inspired by the game Go Wish [16] but contains its own set of cards adapted to the Swiss cultural context and additional rules and content. It can be used with caregivers during structured advance care planning discussions, or it can be proposed as an icebreaker to be played independently at home with loved ones. A printable version of Anticip’action is available in different languages (English, French, German, and Italian) for people who are not at ease with digital tools. The digital version is currently only available in French in Accordons-nous (Textbox 1; Figures 1-6). In this paper, we present the results of the usability test of the digital version of the game.

**Short description of the game rules of Anticip’action.**

**The game is divided into three phases**

1. “Prioritize”—in this phase, the task is to sort 32 cards into 4 categories (“Very important,” “Important,” “Not important,” and “Unsure”). Examples of card statements are “Looking after my appearance” and “Expressing what I couldn’t stand.” Each card includes explanatory text on the back.
2. “Specify”—in this phase, the task is to add personal details to the cards that have been marked as “Very important” and decide whether the topic has already been addressed or still requires action.
3. “Act”—in this phase, the task is to write down actions that could be taken to address the important issues highlighted in the previous phase. To assist in this task, each card suggests possible actions (eg, scheduling an appointment with a health care provider or arranging a meeting with a loved one). The output of the game is a PDF summary of cards ranking, specifications provided during the game, and to-do-list of actions to be done.

## Methods

### Recruitment

The aim was to collect data from 10 participants, as Nielsen and Landauer [17] have demonstrated that a usability test conducted with 10 users can identify >98% of usability issues. The inclusion criteria were as follows: being an adult, having oral and written proficiency in French, and feeling comfortable using a smartphone. A single exclusion criterion was applied: participants should not be familiar with or have previously navigated the Accordons-nous module in the Concerto app. The exclusion criterion was enforced to prevent testers from being biased by prior knowledge that could facilitate ease of use. Overall, the aim was to ensure diversity in gender and age and to include individuals with at least minimal comfort using digital tools. Participants were recruited through the patients-as-partners platform at the HUG as well as via snowball sampling and networking. Participation was voluntary, and participants received an information sheet and consent form in advance. The study was preregistered on the OSF platform [18].

### Ethical Considerations

The research protocol was submitted to the Geneva Commission Cantonale d’Ethique de la Recherche (Req-ID 2024-00994), which determined that the project did not fall within the scope of the Human Research Ordinance and therefore waived the requirement for ethics approval (decision date: September 17, 2024). This study was conducted in accordance with the Declaration of Helsinki. Willing participants were informed of the study aims and procedures and signed an informed consent form. During the data collection and analysis phase, only 3 authors (DC, FE, and CC) had access to the recorded interviews. The recordings were destroyed after analysis. No identifying information was retained in the remaining study files.

### Study Intervention

The entire evaluation was conducted online (via email and videoconference using Zoom [Zoom Video Communications, Inc]). Participants received in advance via email the information sheet and consent form along with instructions on how to join the videoconference with the experimenter and how to install the Concerto app. They were asked not to use the Concerto app before the test and were not informed about the name of the game or where to find it in the app. On the day of the test, the experimenter answered any questions, made sure that informed consent had been signed and returned, and assisted participants in connecting their smartphone to the Zoom videoconference to observe and record their navigation behavior.

The test began with a few demographic and technical questions (age, gender, level of education, type of smartphone used, and smartphone use habits), after which participants were shown a screenshot of the game to gather their first impressions. They then completed a series of think-aloud tasks [19] during which they used the main functions of the game. The 26 tasks were designed to evaluate the usability of the game and to ensure that an average user could navigate effectively through it. Participants were asked to imaging being an individual aged 50 years concerned about illness and advance care directives, who had been advised to use the game Anticip’action. The experimenter then presented the scripted task instructions one by one (Table 1). While completing the tasks, participants were encouraged to verbalize their thoughts, actions, and decisions.

**Table 1:** List and description of the tasks completed during the think-aloud procedure.

Tasks	Expected achievement	Exact wording of the instruction	When the task was considered complete
1. Find and open the game.	Find the game nested in the Accordons-nous module in the Concerto app.	Your friend told you about the game. He told you that the game is in Concerto. You are now in Concerto. More precisely, the game is in the Accordons-nous module. Your first task is to find the game and open it. Let’s go!	The participant opens the Concerto app, scrolls to and clicks on the Accordons-nous module, clicks on the “Talk about it” menu at the bottom of the screen to access the game entry point, and then clicks on the “Play” button.
2. Read rules and start.	Understand the logic of the welcome page, which includes 2 buttons: one for accessing the rules of the game and one for starting a new game (Figure 1).	First, read the rules, and start the game.	The participant clicks on the “Game rules” button, reads the rules, closes the rules page by clicking on the X icon, clicks on the “New game” button, reads the pop-up instructions when they appear, and then closes the pop-up.
3. Select “Feelings and relationships.”	Understand that there are 4 thematic card piles that can be opened (Figure 2).	Select the “Feelings and relationships” pile, and open it.	The participant clicks on the third (pink) icon at the bottom of the screen, which represents the “Feelings and relationships” pile.
4. Flip the first card.	Understand that the card can be flipped to obtain more information on the topic (Figure 3).	Flip the first card.	The participant clicks on the “flip card” button at the top of the screen.
5. Scroll through cards.	Understand that the content of the opened pile can be explored by scrolling (Figure 3).	Scroll once to the left and once to the right to see the other cards in this pile.	The participant uses the navigation arrows to scroll through the cards in the pile.
6. Classify as “Unsure.”	Understand the classification system, including the “Unsure” category (Figure 3).	Classify the first card as “Unsure.”	The participant clicks on the rectangular “Unsure” button.
7. Classify as “Very important.”	Understand that the first filled heart icon represents the “Very important” category (Figure 3).	Classify the next 3 cards as “Very important.”	The participant clicks on the filled heart icon.
8. Exit the pile.	Understand how to return to the main screen of the game when a pile is opened (Figure 3).	Exit this pile.	The participant clicks on the X icon on the top right of the screen.
9. Open the “Practical questions” pile.	Identify and open another thematic card pile (Figure 2).	Open the “Practical questions” pile.	The participant clicks on the second (orange) icon at the bottom of the screen.
10. Classify 9 cards as “Very important.”	Learn the rule limiting the number of cards classified as “Very important” to a maximum of 10 (Figure 3)	Classify the first 9 cards as “Very important.”	The participant clicks on the filled heart icon until a pop-up message appears.
11. Read and close pop-up.	Understand the content of an instruction pop-up and close it.	[Pop-up appears] Read this message, and close it.	The participant reads the pop-up message and clicks on either the X icon or the “Understood” button.
12. Find and scroll through surplus cards.	Discover where to find already classified cards (Figures 2 and 4).	Find the surplus cards, and scroll through them.	The participant clicks on the X icon on the top right of the screen to exit the pile, clicks on the “Prioritized cards” button icon on the main screen (top left) to view and update already classified cards, clicks on “Very important,” and scrolls through the cards.
13. Classify surplus cards as “Important.”	Understand how to reclassify cards in another category (Figure 4).	Classify the surplus cards as “Important” and then confirm.	The participant clicks on a card classified as “Very important” and clicks on the half-filled heart icon, repeating the process until task completion.
14. Classify all cards.	Understand that all cards must be classified in the first “Prioritize” phase of the game (Figure 2).	Classify the following cards as “Not important.”	The participant clicks on card piles and clicks on the empty heart icon below each card, repeating the process until no more cards are available.
15. Check card count in each category.	Understand the logic of the 4 classification categories and their associated numbers (located on the main screen; Figure 2).	Do you know how many cards are present in each classification category (“Very important,” “Important,” “Not important,” “Unsure”)?	The participant reads and interprets the classification numbers located under the category’s icons.
16. Go to next phase of the game.	Understand how to go to the second phase of the game.	You can proceed to the next step.	The participant clicks on the “Next step” button (it appears at the bottom of the main screen when all cards have been classified).
17. Read instructions and continue.	Discover the game rules for the next phase (“Specify”) of the game.	[Pop-up appears] Read the instructions, and continue the game.	The participant reads the instructions and closes the pop-up by clicking on either the X icon or the “Understood” button.
18. Add text to a card.	Understand how to add text to a card (Figure 5).	Write comments on the first card by adding text in the designated space (eg, a letter).	The participant clicks on the card pile (which opens the first “Very important” card) and enters text in the designated space on the card.
19. Validate and define whether to act.	Understand that the specifications provided on a card can only be validated after it is additionally specified whether the topic still requires action or has already been addressed (Figure 5).	Validate the card to move on to the next one, knowing that action is still required.	The participant clicks on the second radio button (“I have to take care of this”) and then clicks on the “Validate and classify this card” button.
20. Locate the rules.	Identify how to access the game rules at any time during the game (Figure 2).	Imagine that you suddenly have doubts about some game rules and would like to consult them. Where do you find the rules of the game?	The participant clicks on the X icon to exit the pile and, on the main screen, clicks on the “?” icon (top left); alternatively, the participant quits the game by clicking on the X icon on the main screen and then clicking on the “Game rules” button on the welcome page.
21. Validate as “Resolved.”	Understand that for all cards in the “Specify” phase, a decision has to be made about whether or not it calls for an action (Figure 5).	Validate the following cards (without writing text on them) as “Resolved” and continue the game.	The participant clicks on the pile of cards not yet specified (located at the bottom of the main screen); for each card, the participant clicks on the first radio button (“This has been taken care of”) and then clicks on the button “Validate and classify this card.”
22. Review and check all actions.	Understand how to handle the cards remaining in the final phase (“Act”) of the game: open each card, review the specifications, and choose related actions (Figure 6).	Review your specifications, and check all the actions.	The participant clicks on the pile of cards requiring action (located at the bottom of the main screen), clicks on the “Review my specifications” button, clicks on the “flip card” button again, and clicks on all actions.
23. Add more text to a card.	Understand how to enter one’s own ideas about actions to be taken (Figure 6).	Add text in the designated space (eg, a letter).	The participant clicks in the “Other ideas” textbox and enters text.
24. Validate the card.	Understand how to validate action choices related to a card (Figure 6).	Validate the card.	The participant clicks on the “Validate and classify this card” button.
25. Export results and return to the game.	Understand how to export a summary of the game.	Export your results, and return to the game.	The participant clicks on the “Export game summary” button (it appears when all actions have been defined), reads the data confidentiality pop-up, and clicks on the “Understood” button.
26. Erase data and exit the game.	Understand that a decision can be made to erase or retain the data when exiting the game.	Exit the game, and delete the data.	The participant clicks on the “Quit” button and, when the pop-up appears, clicks on the “Quit and delete my data” gray band.

Finally, posttest questionnaires were administered to gather participants’ subjective evaluation of the usability, attractiveness, and relevance of the game as well as their overall endorsement. The questionnaires included open-ended questions to further explore usability issues (Multimedia Appendix 1). While usability was the primary focus of the study, these additional measures helped provide a broader understanding of users’ perceptions and expectations.

### Measures Used and Data Analysis

#### First Impressions

The 5-second test [20] was used to gather participants’ spontaneous first impressions of the game. In this test, the main screen of the game (Figure 2) was shown for exactly 5 seconds before being replaced by a black screen. Participants were then asked four questions: (1) “What do you remember about the visual elements of the interface?” (2) “What do you think are the objectives of the game?” (3) “What is your overall impression of the game (on a 5-point scale ranging from *very bad* to *very good*)?” (4) “How do you evaluate the aesthetics of the game (on a 5-point scale ranging from *very unsightly* to *very attractive*)?” The 2 open-ended questions were presented first because this has been shown to better support memory recall [21].

#### Think-Aloud Procedure

##### Task Success

Performance was assessed based on whether participants succeeded or failed in completing each task, with or without assistance from the experimenter. The following scoring system was applied: 0=the participant failed, and the experimenter ultimately provided the answer; 1=the participant succeeded but only with the experimenter’s help; 2= the participant succeeded after the experimenter repeated the task instructions (in cases where the participant had forgotten the task; this was not considered assistance); 3=the participant succeeded independently but explored the interface before arriving at the correct result (correct result and path but after some exploration); 4=the participant succeeded without help but did not use the shortest path (correct result via an alternative route); and 5=the participant succeeded without assistance and easily found the shortest path.

##### Clicks to Complete Tasks

The number of clicks required to complete each task was recorded. As shown in Table 1, some tasks required more clicks than others, whereas for certain tasks, multiple response paths were possible.

##### Time Spent on Tasks

The time (in seconds) that participants took to complete each task was recorded. Timing began when the experimenter finished giving the task instruction for the first time and ended when the participant successfully completed the task. Any time spent digressing during the task (eg, making critical comments about the game’s design or sharing a personal memory) was recorded as “digression time” and subtracted from the total time.

##### Analysis of Errors and Problems Encountered

The errors and problems encountered by participants while completing the 26 tasks were recorded and analyzed. Each issue was documented in a Microsoft Excel file; categorized according to the heuristics (such as information density, consistency, and code meaning) of Bastien and Scapin [22]; and assigned a severity score using a 0 to 4 scale based on the recommendations of Nielsen and Landauer [17]: 0=disagreement on whether it constitutes a usability problem; 1=cosmetic issue—need not be fixed unless extra time is available; 2=minor usability issue—fixing this should be given low priority; 3=major usability issue—important to fix and should be given high priority; and 4=usability catastrophe—imperative to fix this before the product can be launched.

##### Data Analysis

To ensure reliability, all authors independently coded 2 recordings. The results were then compared and discussed to resolve discrepancies or doubts about specific interpretations. The first author coded the remaining data independently.

#### Questionnaires

##### Perceived Usability

The French version of the standard System Usability Scale (SUS) was used to evaluate participants’ perceptions of the game’s usability [23]. The SUS provides an indication of the effectiveness, efficiency, and overall ease of use of a digital product. It consists of 10 items rated on a 0 to 4 Likert scale, yielding a score ranging from 0 to 100 (each item contributing up to 10 points). A score of approximately 85 is considered excellent, approximately 72 good, approximately 53 average, and approximately 38 poor.

As discussing end-of-life and advance care planning issues is not an everyday activity, Anticip’action is not expected to be played repetitively. Consequently, the first item of the SUS—“I think I would like to use this system frequently”—is only partially applicable to evaluating the game. To address this, a slightly modified version was added to the original 10 items: “I think that if I need to consider what is important to me at the end of life, I would give priority to this game.” This adjustment allowed for the calculation of 2 SUS scores: the standard version and an adapted version that included the modified item.

##### Perceived Attractiveness

The French version of the standard 28-item AttrakDiff questionnaire [24] was used to evaluate participants’ experience across four dimensions of user experience, each comprising 7 items: (1) pragmatic quality, which measures the product’s usability and the perceived ease with which users can achieve their goals; (2) hedonic quality–stimulation, which assesses the extent to which the product satisfies the need for stimulation by offering new, interesting, and engaging content, features, and interaction styles; (3) hedonic quality–identity, which examines how well the product supports a social function and communicates a sense of user identity; and (4) overall attractiveness, which represents the perceived value of the product based on its pragmatic and hedonic qualities. This questionnaire includes pairs of contrasting adjectives rated on a 7-point scale ranging from −3 to +3. Scores close to the mean (between 0 and 1) indicate that the product has achieved its objectives without any significant negative impact, although improvements could be made to create a more positive user experience or to enhance attractiveness. Scores outside the neutral zone should be interpreted as either positive (from 1 to 3) or negative (from −1 to −3).

##### Perceived Relevance and Endorsement

To assess participants’ perceptions of the app’s impact on user awareness, knowledge, attitudes, intention to change, and likelihood of actual change in advance care planning behavior, the 6 “perceived impact” items (section F) of the validated Mobile Application Rating Scale (MARS) were used [25]. These items yield an overall mean score on a 5-point Likert scale, interpreted as follows: 1=inadequate, 2=poor, 3=acceptable, 4=good, and 5=excellent. To evaluate endorsement, the first 5-point Likert item of the “subjective quality” section (section E) of the MARS was used. This item asks participants whether they would recommend the digital game to others who could benefit from it. This questionnaire was translated into French in accordance with the best practices described by Wild et al [26].

#### Participants’ Qualitative Feedback

At the end of the study, participants were asked 5 open-ended questions to gather their final feedback on the game. These questions aimed to assess (1) what participants appreciated about the game, (2) what aspects they disliked, (3) which tasks they found particularly challenging, (4) what they felt was missing in the game, and (5) whether they had any additional comments to share. To analyze the responses, the guidelines set by Paillé and Mucchielli [27] for qualitative data analysis were followed. Specifically, Excel was used for organizing and working with the material, with integrated in-text notes that were differentiated by color to facilitate categorization. A continuous thematic analysis approach was applied to systematically identify emerging themes and patterns throughout the feedback.

#### Content Readability

To assess the readability of the game’s textual content, all text was compiled into a Microsoft Word document and evaluated using the Scolarius test [28], which was developed by Influence Communication, a Canadian media analysis organization. The readability score is calculated similarly to the Flesch Reading Ease score (commonly used for English-language materials). The test measures word and paragraph length to provide a score ranging from 50 to 250, interpreted as follows: a score between 50 and 89 corresponds to an elementary school reading level, 90 to 119 indicates a high school reading level, 120 to 149 reflects a college reading level, and 150 to >189 represents a university reading level.

## Results

### Recruitment

The procedure was pilot-tested with 2 participants to master the technical aspects of conducting the test online with multiple devices and to refine the multiple task instructions. These data are not included in the results. During September and October 2024, we recruited 12 participants for the online usability test. However, data from 2 participants could not be analyzed due to major technical issues: a failure of the screen-sharing functionality on Zoom in 1 case and bad quality recording in the other. The final dataset includes complete responses from 10 participants.

The mean age of the participants included in the final dataset was 41 (range 25-65) years, and the sample was diverse in terms of gender (men: 5/10, 50%; women: 5/10, 50%) and type of smartphone (operating system) used (Table 2). All participants reported using their smartphones daily, demonstrating familiarity with digital interfaces and technological tools, and they had a rather high level of education. Overall, the sample was skewed toward younger adults, with only 1 (10%) of the 10 participants being aged >60 years. All participants reported at least moderate digital literacy.

**Table 2:** Participants’ characteristics.

Participant	Gender	Age (y)	Education	Smartphone operating system
P1	Woman	54	Professional higher education	iOS
P2	Woman	25	University	iOS
P3	Woman	35	Professional higher education	iOS
P4	Man	53	High school	iOS
P5^a^	Woman	36	High school	iOS
P6	Man	34	High school	Android
P7^a^	Man	59	University	iOS
P8	Man	35	University	Android
P9	Woman	32	University	iOS
P10	Man	27	University	iOS
P11	Man	65	University	iOS
P12	Woman	49	University	iOS

^a^Data from these participants could not be used because of technical issues.

### First Impressions

In the visual recall task, 80% (8/10) of the participants noticed the presence of colored cards. Of these 8 participants, 3 (38%) observed that the cards had titles, and 2 (25%) noted the numbers displayed in the center of each card. The second most frequently recalled element (5/10, 50%) was the instruction bubble positioned in the center of the screen. Finally, the 3 circular button icons representing the game’s 3 phases were remembered by 5 (50%) of the 10 participants; within this group, 3 (60%) also noticed the grayed-out card images, and another 3 (60%) noticed the text beneath the icons. Some elements remained unnoticed, including the gray background of the game; the title of the game phase at the top of the screen; the 2 square buttons on the top left (question mark icon) and top right (X icon) to access the rules or exit the game, respectively; the classification symbols (heart icons and question mark); and the numbers below these symbols. Some participants had false memories. Of the 10 participants, 1 (10%) remembered the presence of a question, possibly confusing it with the instruction bubble in the center of the screen; 1 (10%) reported 4 button icons at the top of the screen (instead of 3); and 1 (10%) mentioned “choices with several options,” possibly referring to the instructions and heart icons. In addition, there was evidence of imprecise recall and potential misinterpretation of certain elements; for example, of the 10 participants, 1 (10%) described a white background for the game, 1 (10%) reported that the colored cards were placed in the center of the screen (instead of at the bottom), and 1 (10%) misremembered the position of the text as being below the cards rather than above them.

Regarding participants’ understanding of the game’s objectives, half (5/10, 50%) were unable to provide an answer, while the other half (5/10, 50%) identified it as a card game. Of those who recognized it as a card game, 60% (3/5) suggested that the objective might involve making choices. Each of these 5 participants provided at least 1 interpretation of the game: asking questions about various hospital-related topics, completing a multiple-choice quiz, associating each card pile with a specific value, obtaining information with the option to change one’s mind, sorting cards by theme, and clicking on heart icons to indicate preferences (this participant seemed to recall the presence of the heart icons although this was not explicitly expressed during the visual recall task).

Participants’ general impression of the game was mixed: of the 10 participants, 4 (40%) rated it as good, 3 (30%) as average, 2 (20%) as bad, and 1 (10%) as very bad.

Their evaluation of the game’s aesthetics was also mixed: of the 10 participants, 1 (10%) found it very attractive, 2 (20%) rated it as attractive, 5 (50%) as neither attractive nor unsightly, 1 (10%) as unsightly, and 1 (10%) as very unsightly.

### Think-Aloud Procedure

#### Task Success

At the analysis stage, task 19 (which overlapped partially with task 21) was excluded from the results because it seemed that most participants failed to understand the instruction due to suboptimal wording. The instruction began with “Validate the card” before unclearly alluding to the constraint that a card can only be validated once a decision has been made about whether one should do something about the card topic. Consequently, participants attempted to validate the card prematurely, before ticking the appropriate box. Unfortunately, this methodological flaw was not identified during the pilot testing phase.

**Figure 7:**
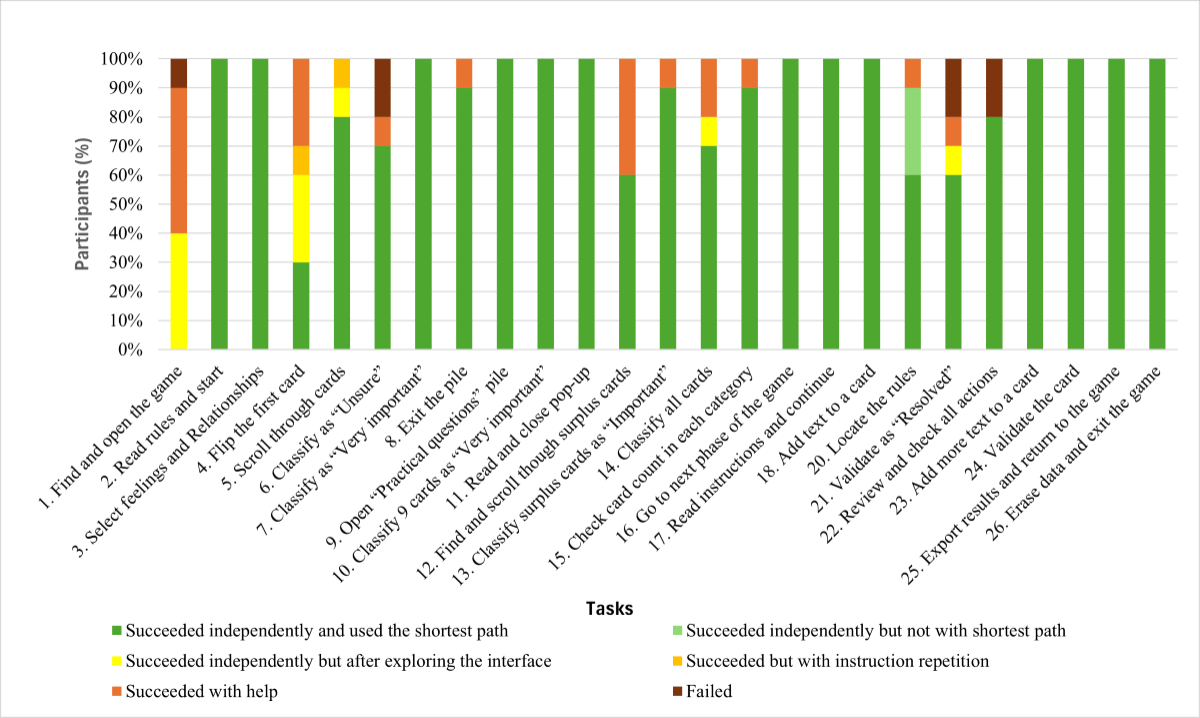
Proportion of participants who succeeded or failed in completing study tasks during the think-aloud procedure, with or without input from the experimenter, the type of input received, and whether the shortest path was used.

As illustrated in Figure 7 and Table S1 in Multimedia Appendix 1, most of the tasks (21/25, 84%) were completed successfully by all participants, while some (4/25, 16%) required assistance. In 83.2% (208/250) of the cases, participants successfully completed tasks without assistance and found the shortest path. In 1.2% (3/250) of the cases, participants succeeded independently but did not follow the most direct route. In 4% (10/250) of the cases, participants explored the interface before finding the correct solution. Meanwhile, 8.8% (22/250) of the cases required intervention from the experimenter. Finally, 2.8% (7/250) of the cases resulted in failure, as participants were unable to complete the tasks even with assistance.

Some tasks required only minimal assistance (eg, tasks 4 and 14), while other tasks presented notable usability issues (Table 3). In particular, for completing task 1—finding the game in the Concerto app—6 (60%) of the 10 participants required assistance, such as instruction repetition, hints, additional information, or the correct answer. For task 4, which involved flipping a card, 6 (60%) of the 10 participants experienced difficulties locating the dedicated button (Figure 3). For task 6—classifying a card in the “Unsure” category—3 (30%) of the 10 participants needed substantial intervention (such as additional information, guiding questions, or answers to questions) to understand that the square “Unsure” button was clickable (the other clickable categories were represented by round buttons; Figure 3). For task 12, which involved finding the surplus cards classified as “Very important,” 6 (60%) of the 10 participants faced difficulties, necessitating multiple forms of assistance, such as instruction repetition, additional information, hints, answers to questions, or guiding questions. The nature of these difficulties indicates that the wording of the task instruction was not optimal: specifically, the phrase “cartes en surplus” (“surplus cards”) used in the instruction could be misleadingly interpreted as the existence of some “extra cards” in the game, although the aim was to convey the information that too many cards had been allocated to the “Very important” category, and some would need to be removed from the category before moving on to the next phase of the game. Task 21 was challenging for 3 (30%) of the 10 participants. It required understanding that in the “Specify” phase of the game, each card needed to be evaluated to determine whether it required further action before it could be marked as “resolved,” after which the participant could proceed to the “Act” phase of the game (Figure 5). However, the task instruction was short and allusive, which could explain the participants’ need for more guidance. In task 22, of the 10 participants, 1 (10%) failed to understand that the pile in the center needed to be “resolved,” and 1 (10%) failed to click on the “Review my specifications” button to check the specifications related to the card (Figure 6). In both cases, the experimenter provided the answer.

**Table 3:** Description of the problems encountered by participants (n=10) during the think-aloud tasks.

Tasks and problems encountered by participants		Frequency, n (%)	Category (according to the heuristics of Bastien and Scapin [22])	Severity (0 to 4 scale based on the recommendations of Nielsen and Landauer [17])
**1. Find and open the game.**				
	1.1. Could not find the game and clicked on the wrong tab	3 (30)	Guidance: grouping and distinction	4
	1.2. Could not find the game: did not see the menu at the bottom	2 (20)	Guidance: grouping and distinction; consistency	4
	1.3. Thought they were already in the game when they were on the wrong page in Accordons-nous	2 (20)	Guidance: prompting; workload: information density	3
	1.4. Complained about too much text	1 (10)	Workload: information density	1
	1.5. Did not see the “play” button, which allows entry into the game	1 (10)	Guidance: legibility	3
	1.6. Misunderstood the “Anticip’action” tab as a way to enter the game, although it is actually a tab to navigate within sections in the Accordons-nous module	2 (20)	Guidance: grouping and distinction	3
**2. Read rules and start.**				
	2.1. Felt overwhelmed by the text	2 (20)	Workload: information density	1
**4. Flip the first card.**				
	4.1. Did not see the button to flip the card	4 (40)	Guidance: grouping and distinction; compatibility	3
	4.2. Interpreted the return icon as a door to return to the menu	1 (10)	Consistency	1
	4.3. Interpreted the card title as a clickable button	1 (10)	Consistency	0
**6. Classify as “Unsure.”**				
	6.1. Used drag and drop to classify a card (which is not a function in the game)	2 (20)	Consistency and compatibility	1
	6.2. Clicked on the wrong button	2 (20)	Consistency and significance of codes	2
**8. Exit the pile.**				
	8.1. Did not see the X icon (located on the top right of the screen) to exit the pile	1 (10)	Guidance: prompting	0
**12. Find and scroll through surplus cards.**				
	12.1. Tried to find the extra cards by clicking on the heart icons on the main page (which is not a function in the game)	3 (30)	Guidance: prompting and grouping and distinction; significance of codes	3
	12.2. Could not find the extra cards after exploring the screen	6 (60)	Guidance: grouping and distinction; consistency	3
**13. Classify surplus cards as “Important.”**				
	13.1. Did not see the confirmation button after completing the task	1 (10)	Workload: minimal actions; adaptability: flexibility	2
**18. Add text to a card.**				
	18.1. Did not understand that to validate the written text, one needs to click on the X icon	1 (10)	Guidance: prompting; compatibility	2
	18.2. Clicked in the text area when only the written area was active	1 (10)	Compatibility	1
**20. Locate the rules.**				
	20.1. Did not see the button to find the rules	3 (30)	Guidance: legibility	1
	20.2. Did not want to exit the game for fear of losing data	1 (10)	Error management: error protection; workload: information density	2
**21. Validate as “Resolved.”**				
	21.1. Did not click on the card pile in phase 2 to continue the game	1 (10)	Guidance: grouping and distinction; workload: information density	0
**22. Review and check all actions.**				
	22.1. Clicked on the specified cards button instead of the blue pile at the bottom	1 (10)	Guidance: prompting; significance of codes	1
	22.2. Did not see the button to review the details	2 (20)	Guidance: immediate feedback and legibility; significance of codes	3

#### Clicks to Complete Tasks

As shown in Table 4, a comparison between participants’ actual number of clicks and the expected optimal number reveals varying levels of efficiency across tasks. For most of the tasks, participants generally stayed close to the expected optimal number of clicks, indicating a solid understanding of the interface and task requirements. However, several tasks stand out with higher click counts, suggesting areas where participants may have encountered challenges or required additional exploration before completing the task. In particular, task 1, which involved finding the game in the Concerto app, was completed with a mean of 10.30 (SD 4.47) clicks compared to the expected 3.

**Table 4:** Participants’ overall click performance per task: mean number of clicks used, expected optimal number of clicks, and deviation from the optimal number.

Tasks	Number of clicks, mean (SD)	Optimal number of clicks, n	Difference
1. Find and open the game.	10.30 (4.47)	3.00	7.30
2. Read rules and start.	4.00 (0.00)	4.00	0.00
3. Select “Feelings and relationships.”	1.00 (0.00)	1.00	0.00
4. Flip the first card.	2.50 (1.43)	1.00	1.50
5. Scroll through cards.	3.00 (1.70)	2.00	1.00
6. Classify as “Unsure.”	2.40 (2.84)	1.00	1.40
7. Classify as “Very important.”	3.20 (0.42)	3.00	0.20
8. Exit the pile.	1.20 (0.63)	1.00	0.20
9. Open “Practical questions” pile.	1.00 (0.00)	1.00	0.00
10. Classify 9 cards as “Very important.”	8.10 (0.32)	8.00	0.10
11. Read and close pop-up.	1.00 (0.00)	1.00	0.00
12. Find and scroll through surplus cards.	5.20 (2.30)	4.00	1.20
13. Classify surplus cards as “Important.”	3.40 (0.97)	3.00	0.40
14. Classify all cards.	25.40 (0.97)	25.00	0.40
15. Check card count in each category.	0.00 (0.00)	0.00	0.00
16. Go to next phase of the game.	1.00 (0.00)	1.00	0.00
17. Read instructions and continue.	1.00 (0.00)	1.00	0.00
18. Add text to a card.	2.70 (0.95)	2.00	0.70
20. Locate the rules.	2.40 (0.52)	2.00	0.40
21. Validate as “Resolved.”	20.30 (2.26)	19.00	1.30
22. Review and check all actions.	6.30 (3.13)	5.00	1.30
23. Add more text to a card.	2.40 (0.70)	2.00	0.40
24. Validate the card.	1.00 (0.00)	1.00	0.00
25. Export results and return to the game.	3.00 (0.00)	3.00	0.00
26. Erase data and exit the game.	2.00 (0.00)	2.00	0.00

#### Time Spent on Tasks

**Figure 8:**
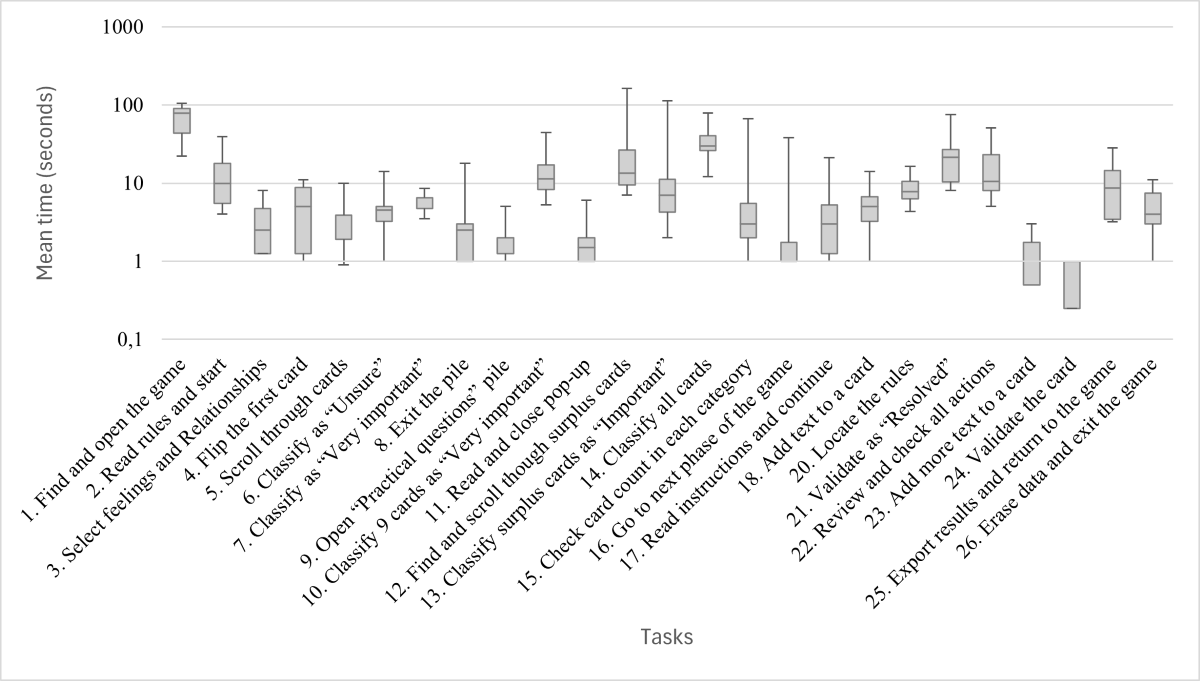
Mean time (in seconds) spent by participants to complete each of the 25 tasks during the think-aloud procedure, displayed on a logarithmic scale.

As illustrated in Figure 8, the analysis of time allocation per task reveals substantial variability among participants. Nevertheless, some patterns and deviations from precalculated optimal times provide insight into specific usability challenges. Notably, task 1 (“Find and open the game”) required considerably more time than anticipated, indicating that participants faced initial difficulties with the game’s discoverability. Participants 6 and 8 displayed markedly extended times for task 1, suggesting that the entry point for locating the game lacked intuitive visibility or clarity. Divergence from the optimal could also be observed in tasks 10, 14, and 21, which required participants to categorize or “resolve” numerous cards. By design, these tasks were lengthy rather than difficult to navigate: while some participants elected to read each card before sorting it, others proceeded with the task more quickly without detailed review, leading to substantial individual variations in completion time. A similar pattern could be observed with task 2, which asked participants to read the rules of the game and start: some participants took much more time to read the rules than others. The time taken to complete tasks 12 and 21 (discussed previously) also diverged notably from optimal times, partly due to issues stemming from unclear instructions and partly due to suboptimal guidance in the game.

#### Analysis of Errors and Problems Encountered

Following the criteria formulated by Bastien and Scapin [22], we recorded 23 problems encountered by the participants while completing the 26 tasks (Table 3). Of these 23 problems, 2 (9%) were classified as usability catastrophes, 7 (30%) as major usability problems, 5 (22%) as minor usability problems, 6 (26%) as cosmetic issues, and 3 (13%) as not constituting a usability problem. A majority of the problems (14/23, 61%) were related to guidance difficulties, such as the visibility of visual items or the way they were displayed in relation to one another.

### Questionnaires

#### Perceived Usability

**Figure 9:**
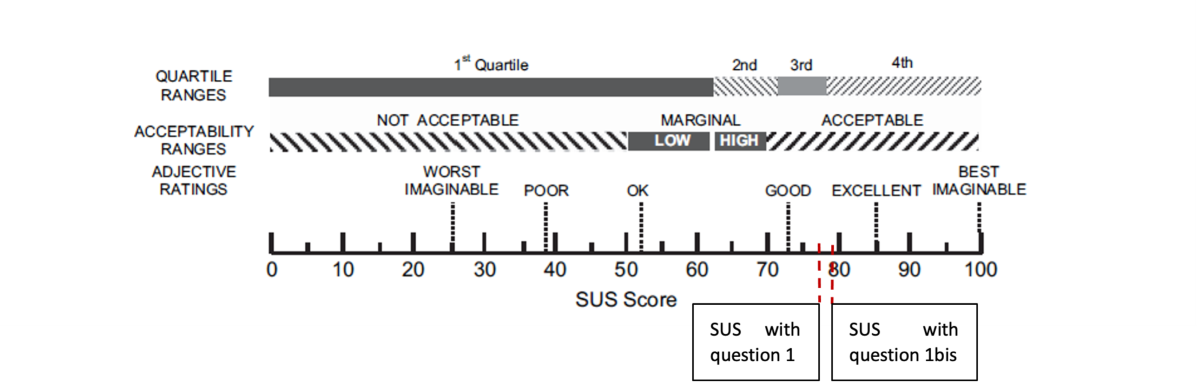
Scores of Anticip’action on the standard and adapted versions of the System Usability Scale (SUS).

As illustrated in Figure 9, the mean score on the standard SUS was 77.75 (SD 5.1) out of 100. The adapted version of the SUS, which included a more fitting variant of the first question, yielded a slightly higher mean score of 79.25 (SD 5.6) out of 100. These results indicate that participants rated the game’s usability between good and excellent.

#### Perceived Attractiveness

**Figure 10:**
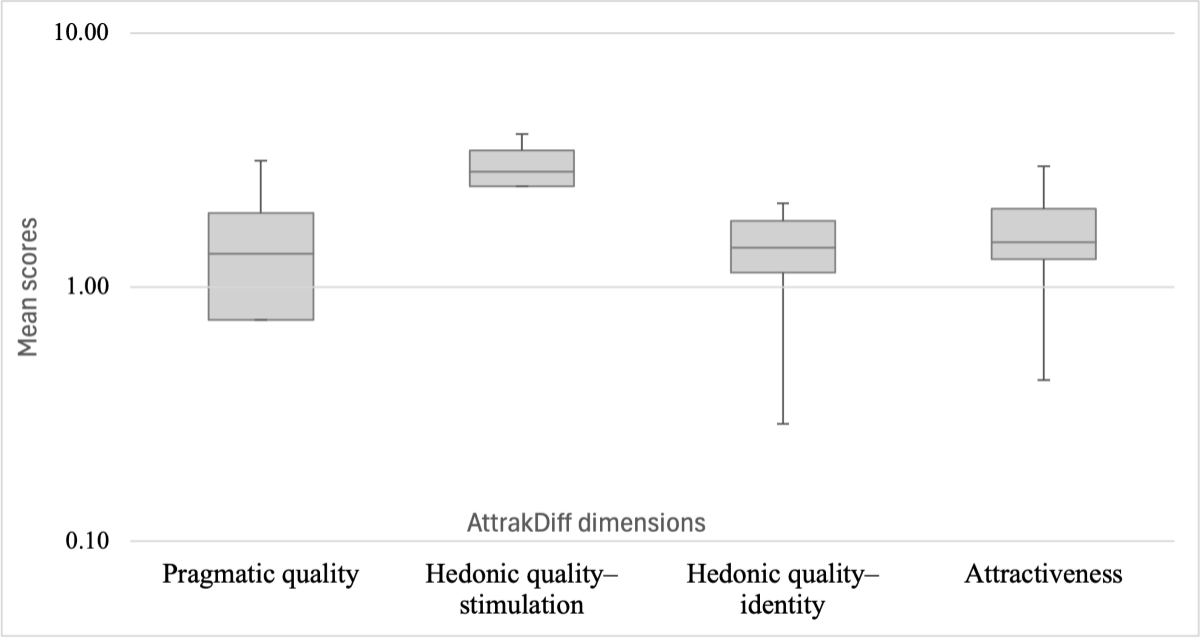
Mean scores of Anticip’action on the 4 dimensions of the AttrakDiff questionnaire, displayed on a logarithmic scale.

The game obtained an overall attractiveness score of 1.57, indicating a positive assessment. As illustrated in Figure 10, positive results were obtained on all 4 dimensions of the test, with scores ranging from 1.16 to 1.57.

#### Perceived Relevance and Endorsement

The mean score on the MARS questionnaire was 4.1 (SD 2.1), indicating that participants evaluated the relevance of the game as good. As illustrated in Table 5, participants were mostly convinced that the game can increase awareness, knowledge, and intention to change regarding the topic of anticipating difficult situations that may arise in the future.

The game also ranked positively on the “I would recommend” question, with a mean score of 3.3 (SD 1.1). Of the 10 participants, 2 (20%) indicated that they would highly recommend the game to everyone who could benefit from it, 1 (10%) would recommend it to many people, 5 (50%) to several people, and 2 (20%) to very few people. No participant stated that they would not recommend the game.

**Table 5:** Average responses of the 10 participants to the Mobile Application Rating Scale (MARS) questionnaire, section F. All questions ended with “[...] the anticipation of difficult situations that may arise in the future.”

	Scores, mean (SD)
Awareness: this game is likely to increase awareness of the importance of...	4.5 (2.4)
Knowledge: this game is likely to increase understanding of...	4.2 (1.9)
Attitude: this game is likely to change attitudes toward improving...	3.8 (1.9)
Intention to change: this game is likely to increase motivation to address...	4.1 (1.4)
Help seeking: use of this game is likely to encourage further help seeking...	4.0 (2.6)
Behavior change: use of this game is likely to increase...	4.0 (2.7)

### Participants’ Qualitative Feedback

Participants generally provided positive feedback about the game. Most found it user-friendly, with clear visuals and easy navigation. Of the 10 participants, 7 (70%) highlighted that the game addressed the sensitive topic of advanced care directives and end-of-life care in an approachable and meaningful way, while 6 (60%) stated that the game encouraged reflection on complex health-related issues and promoted awareness of often-overlooked topics.

Areas for improvement were also suggested. Several participants reported feeling overwhelmed by the amount of information and recommended adjustments to reduce cognitive load. They suggested adding an onboarding tutorial to help users understand the game’s mechanics. Some participants proposed that offering personalized content options (eg, an option to filter cards based on user profile) may help to better tailor the game to individual users’ needs. Navigation issues were also mentioned: participants indicated that the “Unsure” and the “flip card” buttons were not visually intuitive as clickable elements, and navigation could be improved through clearer visual cues such as more prominent buttons. Moreover, some participants recommended incorporating dynamic features such as animations, which could increase accessibility and maintain user engagement. Finally, several participants noted that the term “jeu” (“game”) does not fit with the seriousness of the content and suggested some rephrasing or providing a content advisory to prepare users for the sensitive nature of the topics discussed.

### Content Readability

The readability analysis of the game cards, assessed using the Scolarius program, yielded distinct scores for the rectos (card titles) and versos (explanatory text). The rectos had an average score of 50.5, indicating content accessible to individuals with an elementary school education. By contrast, the versos scored 119.8, which corresponds to a high school reading level. Specifically, in terms of readability, 61% (20/33) of the verso cards were at the elementary school level, 18% (6/33) at the high school level, and 21% (7/33) at the university level.

### Modifications to Be Made Based on the Results

Analysis of the 23 usability issues and the proposed solutions (Table 6) showed that most problems were related to users’ difficulty in identifying which visual elements were actionable. Some buttons were not consistently interpreted as clickable, while certain titles were occasionally mistaken for buttons. No substantial structural modifications seem to be required to address these usability issues, with the exception of the location of the game: it is currently nested within a section of a module (Accordons-nous) inside an app (Concerto), making it difficult for users to access Anticip’action.

**Table 6:** Changes to be made to the game in response to the identified usability issues.

Items	Problems encountered by participants	Changes to be made
1.1, 1.2, and 1.3	Could not find the game nested in the Accordons-nous module	Create a video explaining where the game is located
1.4	Complained about too much text	No changes to be made
1.5 and 1.6	Did not see the “play” button, which allows entry into the game	Highlight the button by bolding the text
2.1	Felt overwhelmed by the text	No changes to be made
4.1	Did not see the button to flip the card	Move the button to the corner or bottom of the card
4.2	Interpreted the return icon as a door to return to the menu	No changes to be made
4.3	Interpreted the card title as a clickable button	Redesign the title by, for example, removing the border
6.1	Used drag and drop to classify a card (which is not a function in the game)	No changes to be made
6.2	Clicked on the wrong button	Align the 4 buttons on the same level, enlarge the question mark, ensure that it uses the same visual weight and style as the heart buttons (eg, same height, rounded shape, and icon structure). Alternatively, add a heart icon with a question mark in the center and place the phrase “I am unsure” below it.
8.1	Did not see the X icon (located on the top right of the screen) to exit the pile	No changes to be made
12.1 and 12.2	Could not find the extra cards	Gray out the hearts or make them clickable to access the menu
13.1	Did not see the confirmation button after completing the task	Change the button design, round the edges, and ensure consistency
18.1	Did not understand that to validate the written text, one needs to click on the X icon	Add a button to validate the text
18.2	Clicked in the text area when only the written area was active	No changes to be made
20.1	Did not see the button to find the rules	No changes to be made
20.2	Did not want to exit the game for fear of losing data	Specify more clearly that exiting the game will not result in data loss
21.1	Did not click on the card pile in phase 2 to continue the game	No changes to be made
22.1	Clicked on the specified cards button instead of the blue pile at the bottom	No changes to be made
22.2	Did not see the button to review the details	Redesign the button, which resembles the card titles too closely, to make it more visible

## Discussion

### Principal Findings

This study evaluated the usability, readability, and user experience of the digital version of the game Anticip’action, designed to facilitate end-of-life discussions and advance care planning decisions. The findings indicate overall satisfactory results.

After 5 seconds of visual exposure to the main screen of the game, participants were able to recall key visual elements, such as the colored cards and accompanying text. However, they struggled to notice other important interface components, including classification symbols and navigation buttons. Some misinterpretations of visual elements were observed, which was confirmed by participants’ fragmented and diverse understanding of the game’s objectives. Moreover, the aesthetics of the game left participants mostly indifferent. These results indicate that there is room for improvement in the visual appearance of the game to better engage users. However, these findings should be interpreted in light of the fact that in a real-world scenario, before reaching the main game screen used for the 5-second test, users would always receive preliminary information about the game’s content, purpose, and rules. Specifically, the welcome page included a pictogram with cards and introductory text explaining the game’s purpose (Figure 1). Moreover, a pop-up explained the card sorting task and classification symbols at the start of a new game.

The think-aloud procedure yielded a high overall success rate. In 83.2% (208/250) of the cases, participants completed the tasks without assistance. Participants’ performance also closely matched the expected number of clicks, indicating that the game offered clear navigation paths. However, for some tasks (4/25, 16%), participants required assistance, revealing serious usability problems. Analysis of the 23 failures and difficulties encountered revealed that 3 (13%) issues were partly due to a suboptimal wording of task instructions (tasks 12 and 21), that 14 (61%) problems were related to guidance, and that there were 9 (39%) major usability issues that needed to be addressed (Table 3). Specifically, the following tasks highlighted important difficulties related to navigation and discoverability: task 1, which involved finding the game within the Concerto app; and task 12, which involved finding and reclassifying the surplus cards classified in the “Very important” category. About half of the participants needed guidance and took longer than the expected optimal time to complete these tasks (Figure 7; Table 7). These issues need to be addressed with clearer instructions and improved visual guidance. Moreover, the test revealed occasional design inconsistencies (eg, some buttons were not visible enough in tasks 1, 4, and 13), excessive cognitive load (eg, some pages had numerous elements and text), and inadequate error prevention (eg, once players opened a pile of cards, they expected to navigate by scrolling, whereas “resolving” cards required clicking on a button; task 21). All identified usability issues could be addressed through minor modifications, as highlighted in Table 6.

**Table 7:** Summary of relevant usability problems encountered during the think-aloud test.

Tasks	Difference between the average time spent by participants per task and the expected optimal time (seconds)	Problems (n=23), n (%)	Participants affected (n=10), n (%)
1. Find and open the game.	29	6 (26)	7 (70)
2. Read rules and start.	7	1 (4)	2 (20)
4. Flip the first card.	2	3 (13)	5 (50)
6. Classify as “Unsure.”	2	2 (7)	3 (30)
8. Exit the pile.	3	1 (4)	1 (10)
12. Find and scroll through surplus cards.	16	2 (7)	5 (50)
13. Classify surplus cards as “Important.”	16	1 (4)	1 (10)
18. Add text to a card.	4	2 (7)	2 (20)
20. Locate the rules.	4	2 (7)	4 (40)
21. Validate as “Resolved.”	8	1 (4)	1 (10)
22. Review and check all actions.	5	2 (7)	3 (30)

At the end of the test, Anticip’action was rated between good and excellent on the SUS. It obtained a mean score of 77.75 out of 100 on the standard SUS and a mean score of 79.25 out of 100 on the adapted version. The increase in score resulting from the adaptation of the first question highlights the importance of context-specific adjustments to usability assessment tools.

The game also received an overall positive assessment of its attractiveness, although there remains room for improvement. The global score of 1.57 on the AttrakDiff questionnaire reflects a generally positive perception. Scores on the pragmatic and hedonic scales ranged from 1.16 to 1.57, all above zero, indicating a baseline level of user satisfaction. Hedonic quality received the lowest score, suggesting that improvements are needed to better engage users and sustain their interest. Enhancements to the design and animated and visually attractive content, such as the inclusion of more visually dynamic elements in the game, could increase its attractiveness while addressing some navigation or cognitive load issues. Reducing the number of actions required in certain steps, notably for re-sorting the cards or “resolving” them in the “Specify” phase of the game, could help maintain user engagement. At a minimum, greater clarity and streamlining of these task processes would improve user experience.

Although this test included a small number of participants and no quantitative conclusions should be drawn, we noticed that older participants and those recruited via the patients-as-partners program at the hospital rated the game more positively. This may be due to a higher baseline receptivity to the topic of advance care planning discussions.

Anticip’action was also evaluated as relevant, with an overall score of 4.1 out of 5 on the MARS. Specifically, the game was judged as particularly impactful in raising awareness, improving knowledge, and motivating behavior change regarding advance care planning. Participants also endorsed the game with a mean score of 3.3 out of 5 on the “I would recommend” question. Given the specificity and sensitivity of the topic, this result is promising.

The readability analysis showed that the card titles are understandable for individuals with an elementary school education, which is an excellent result. However, the longer explanations on the versos of the cards require a high school reading level, indicating room for improvement. Part of the difficulty may be due to the use of gender-neutral language (which has the effect of increasing the lengths of sentences in French) as well as to the use of unavoidable technical terms such as *surrogate decision maker*, *digital legacy*, or *lasting power of attorney*. Efforts are underway to streamline and simplify the card content while preserving its informational value.

Participants provided overall positive qualitative feedback while pointing out aspects that could be improved. Most described the game as user-friendly, with clear visuals and easy navigation. They highlighted its relevance in fostering engagement and encouraging reflection on complex topics such as advance care directives.

However, some participants identified areas that needed improvement. Their concerns included the cognitive overload and some unclarity in the navigation process related to specific tasks. Regarding cognitive overload, some parts of the game contained dense textual information. Although the game achieved good usability scores overall, this suggests that some refinement may be necessary to improve accessibility, for instance, through progressive disclosure, pictorial supports, or guided onboarding elements. Some participants emphasized the need for an onboarding tutorial to improve accessibility as well as more animations, intuitive interactive elements, clearer buttons, and personalized content pathways to improve visual clarity and user engagement. Some participants also disagreed with the label “game” for such a conversation tool and expressed the need for providing a content advisory to prepare users for the sensitive nature of the topics discussed. This highlights a semantic tension between the playful connotation of the term “game” and the seriousness of the topic, which may require careful framing to ensure user acceptance.

To address the identified issues while taking participants’ recommendations into account, several improvements are envisaged. Easily implementable solutions are detailed in Table 6. Furthermore, modifications may include the introduction of an onboarding tutorial to enhance initial navigation and reduce cognitive load. Options to personalize some features of the game (eg, relaxing the 10-card limit) or to filter subgroups of cards may also help further align the game with diverse user needs and preferences. In addition, enhancing the game’s visual appeal by incorporating more dynamic features (eg, animated movement of cards between piles) could increase engagement and satisfaction, especially among younger users.

### Limitations

Although this study reports positive usability results, it does not demonstrate that Anticip’action is an effective tool to promote end-of-life discussions. Moreover, the quantitative results from the SUS, AttrakDiff, and MARS questionnaires should be interpreted with caution, given the limited number of participants (n=10). Complementary studies with appropriate designs and more diverse populations are necessary to confirm the game’s perceived value and effectiveness.

One important limitation of this study is the demographic profile of the participants. The sample included 10 individuals of different ages (ranging from 25 to 65 y), of whom 6 (60%) were in the younger-to–middle adult range (25-45 y). Prior research has shown that age can influence usability metrics, including task performance and perceived ease of use [29]. The limited sample size in this study does not allow for an evaluation of this effect. Moreover, the sample included a participant aged >60 years and none with low digital literacy or lower levels of education. As such, the findings may not be generalizable to these groups. Usability outcomes may differ significantly among older adults or individuals with low digital literacy, who may face greater barriers in navigating the tool. This possibility should be considered when interpreting our findings. Future work should explicitly include a more diverse participant pool, encompassing a wider range of health statuses and cultural backgrounds, to evaluate the accessibility and acceptability of the tool in broader populations.

Participants were asked to imagine being an individual aged 50 years reflecting on end-of-life preferences. This scenario was used to standardize the testing context, but it does not replace real-life engagement with the topic. Future studies should include participants who are directly involved in advance care planning processes.

The test was conducted online with the game displayed on participants’ devices. Therefore, their facial expressions could not be observed and used as nonverbal cues to assess the gravity of usability issues.

Part of the participants’ failure to complete some tasks (in particular, task 19, which was removed from the analysis, as well as tasks 12 and 21) may be due to suboptimal wording of the instructions.

Some participants took more time than others to read the text on the cards before completing the tasks. This difference in participants’ behavioral style could have influenced the time spent on tasks, although it is not an indication of failure to complete them. This was particularly obvious on task 14 (“Classify all cards”) and may have also affected tasks 10 and 21. Therefore, time-based measures are difficult to interpret as usability factors in this study.

### Conclusions

Anticip’action is a conversation tool aimed at initiating end-of-life and advance care planning discussions and increasing patients’ empowerment during these discussions. It offers accessible content, simple navigation, and thoughtful discussion prompts to support individuals, families, and health care professionals in exploring these important and often challenging topics. It is also designed to motivate related action planning, such as writing advance directives.

This study evaluated the usability, readability, and user experience of the digital version of the game with participants of different ages (ranging from 25 to 65 y) who were already familiar with digital interfaces and technological tools. The results demonstrated that participants found the game generally user-friendly, with clear visuals and intuitive navigation. Qualitative feedback highlighted its relevance. Specific challenges were identified, including cognitive overload and some unclear navigation elements. Addressing these issues through improved design, clearer instructions, and better user guidance would further enhance user experience and engagement.

Overall, the usability findings validated Anticip’action as an accessible conversation game for patients who are minimally at ease with digital tools and the French language. While additional testing with health care professionals and broader user groups (including older patients and individuals less comfortable with digital solutions) would strengthen its evidence base, our findings indicate that Anticip’action could be a valuable resource for some patients, families, and caregivers navigating end-of-life decisions and advance care planning. It would be interesting to translate it into other languages, as is the case for the printed version of Anticip’action.

## Appendix

Multimedia Appendix 1 Table showing task completion success rates and related details, along with the questionnaires and the discussion grid used for the final qualitative feedback, provided in both the original French and English translation.

## Abbreviations

HUG: Geneva University Hospitals
MARS: Mobile Application Rating Scale
SUS: System Usability Scale

## Data Availability

The datasets generated and analyzed during this study are available in the Open Science Foundation (OSF) repository [30].
