# Supplementary material for: A Digital Serious Game (Anticip’action) to Support Advance Care Planning Discussions in the General Population: Usability Study
Source: JMIR Aging. 2025 Aug 21;8:e73378. doi: 10.2196/73378 (PMC12411793; doi:10.2196/73378)
Supplement: Multimedia Appendix 1 [file aging_v8i1e73378_app1.docx]

# Supplementary Information

**Anticip’action A Digital Serious Game to Support Advance Care Planning Discussions in the General Population: Usability Study**

Contents

[Supplementary Information 1](#_Toc200528733)

[Supplementary Table 1](#_Toc200528734)

[Questionnaires Used (in French) 4](#_Toc200528735)

[English Translation of Questionnaires 9](#_Toc200528736)

## Supplementary Table

**Table S1**. Task completion success rates and type of experimenter’s intervention during the think-aloud procedure.

| **Task** | **Success** | **Experimenter’s intervention** |
| --- | --- | --- |
|  | 0= failed  1= succeeded with help  2 = succeeded but with repetition  3 = succeeded alone but exploring before  4 = succeeded alone but not with the shortest path  5 = succeeded alone and used the shortest path | 1 = information: e.g., explanation of how the participant arrived at their current position, or why the module contains certain content,  2 = question: e.g., prompting further exploration "where will you go now?",  3 = answer: if the participant asks a question related to exploration,  4 = repeat the task (not in case of understanding, but as help in solving),  5 = give a hint, 6 = other. Providing the answer to the task is not considered as help because it implies the end of the task. |
|  |  |  |
| 1. Find and Open the Game | 1/10 failed, 5/10 succeeded with help, 4/10 succeeded alone but exploring before | 6/6 hint, 4/6 repeat, 1/6 information |
| 2. Read Rules and Start | 10/10 succeeded alone with shortest path | / |
| 3. Select Feelings & Relationships | 10/10 succeeded alone with shortest path | / |
| 4. Flip the First Card | 3/10 succeeded with help, 1/10 succeeded with repetition, 3/10 succeeded alone but exploring before, 3/10 succeeded alone with shortest path | 6/6 repeat |
| 5. Scroll Through Cards | 1/10 succeeded but with repetition, 1/10 succeeded alone but exploring before, 8/10 succeeded alone with shortest path | / |
| 6. Classify as "Unsure" | 2/10 failed, 1/10 succeeded with help, 7/10 succeeded alone with shortest path | 1/3 information, 1/3 question, 1/3 answer |
| 7. Classify as "Very Important" | 10/10 succeeded alone with shortest path | / |
| 8. Exit the Pile | 1/10 succeeded with help, 9/10 succeeded alone with shortest path | 1/1 repeat |
| 9. Open Practical Questions Pile | 10/10 succeeded alone with shortest path | / |
| 10. Classify 9 Cards as "Very Important" | 10/10 succeeded alone with shortest path | / |
| 11. Read and Close Pop-Up | 10/10 succeeded alone with shortest path | / |
| 12. Find and Scroll Surplus Cards | 4/10 succeeded with help, 6/10 succeeded alone with shortest path | 4/4 info, 1/4 question, 2/4 answer to question, 4/4 repeat, 2/4 hint |
| 13. Classify Surplus Cards as "Important" | 1/10 succeeded with help, 9/10 succeeded alone with shortest path | / |
| 14. Classify all cards | 2/10 succeeded with help, 1/10 succeeded alone but exploring before, 7/10 succeeded alone with shortest path | 2/2 information, 1/2 question, 1/2 answer to question, 2/2 repeat |
| 15. Check Card Count in Each Category | 1/10 succeeded with help, 9/10 succeeded alone with shortest path | / |
| 16. Go to Next Phase of the Game | 10/10 succeeded alone with shortest path | / |
| 17. Read Instructions and Continue | 10/10 succeeded alone with shortest path | / |
| 18. Add Text to a Card | 10/10 succeeded alone with shortest path | / |
| 20. Locate the Rules | 1/10 succeeded with help, 3/10 succeeded alone but not with shortest path, 6/10 succeeded alone with shortest path | 1/1 answer to question |
| 21. Validate as "Resolved" | 2/10 failed, 1/10 succeeded with help, 1/10 succeeded alone but exploring before, 6/10 succeeded alone with shortest path | 1/3 information, 1/3 question, 1/3 answer to question, 1/3 repeat, 1/3 hint |
| 22. Review and Check All Boxes | 2/10 failed, 8/10 succeeded alone with shortest path |  |
| 23. Add More Text to a Card | 10/10 succeeded alone with shortest path | / |
| 24. Validate the Card | 10/10 succeeded alone with shortest path | / |
| 25. Export Results and Return | 10/10 succeeded alone with shortest path | / |
| 26. Erase Data and Exit the Game | 10/10 succeeded alone with shortest path | / |

| **Questionnaire de début** | | |
| --- | --- | --- |
| N° | Question | Options de réponse |
| 1 | Gender | Femme; Homme; Autre |
| 2 | Age | Number |
|  | Quel est le plus haut niveau de formation scolaire que vous avez atteint? | École primaire (école obligatoire); Apprentissage professionnel; Gymnase (collège); Maturité professionnelle; Haute école spécialisée (HES); Université ou EPFL |
| 3 | Quel type de smartphone possédez-vous actuellement ? | IOS; Android; Autre; Je ne sais pas; Je n’ai pas de smartphone |
| 4 | À quelle fréquence utilisez-vous votre smartphone ? | Tous les jours; Plusieurs fois par semaine; Plusieurs fois par mois; Quelques fois par année; Jamais |
| 5 | Avez-vous déjà passé un test utilisateur ? voici comme cela va se passer… | Oui ; Non ; Autre |
| 6 | Le but n’est pas de vous évaluer mais d’évaluer si le jeu est facile à utiliser.  Il est normal que certaines tâches soient difficiles à réaliser. Vous aurez un temps suffisant pour faire chaque tâche et je suis là pour vous aider et vous guider. Faites ces tâches normalement, comme si vous les faisiez pour vous.  Je vais vous demander de penser à voix haute, d’exprimer tout ce qui vous passe par la tête pendant que vous effectuez les différentes tâches.  Est-ce que vous avez des questions ? | Texte libre |
| **Test des 5 secondes** | | |
| Dans un premier temps, l’image d’une étape du jeu vous sera présentée pendant 5 secondes. Ensuite, dans un second temps, plusieurs questions vous seront posées pour recueillir vos impressions vis-à-vis de cette page. Nous vous demanderons d'y répondre le plus sincèrement possible. Cela ne vous prendra pas plus de 5 minutes. Quand vous vous sentez prêt(e), vous pouvez l’expliciter oralement et je cliquerai sur le bouton "Commencer" ci-dessous. | | |
| N° | Question | Options de réponse |
| 1 memory dump question | Quels éléments de l’interface avez-vous retenus (texte, logo, couleur, images, menus...)? | Texte libre |
| 2 Target identification questions | Selon vous, quels sont les objectifs visés par ce système (jeu)? | Texte libre |
| 3 attitudinal question | De manière générale, quelle est votre impression vis-à-vis de ce système (jeu) ? | Très mauvaise; Mauvaise; Moyenne; Bonne; Très bonne |
| 4 attitudinal question | De manière générale, comment jugez-vous l’esthétique de ce système (jeu) ? | Très laide; Laide; Ni laide ni belle; Belle; Très belle |

## Questionnaires Used (in French)

| **Scénarios (think aloud)** | | | |
| --- | --- | --- | --- |
| Vous êtes un homme/femme de 50 ans et venez de rencontrer votre sœur, malade depuis longtemps. La discussion avec elle vous amène à réfléchir au futur, mais vous ne savez pas trop définir vos pensées relatives aux directives anticipées. Ainsi, vous en discutez avec un ami, qui vous conseille d’essayer le jeu Anticip’action situé dans l’app Concerto des HUG. | | | |
| Tâche | | Inputs (a-exploration ; b-indice; ca-réponse)   1. Oui, il y a plein de choses sur cet écran. Est-ce que vous arrivez à... + répéter la tâche. 2. Je vous vois en difficulté et c’est compréhensible. Je peux vous donner un indice. 3. Ce n’est pas si simple. Essayez de cliquer ici, puis là. On est arrivé! Bravo! Passons maintenant à la tâche suivante.   **Phrase de recadrage s’ils digressent trop**: Vous avez encore d’autres tâches à effectuer et je ne veux pas abuser de votre temps. Etes-vous d’accord de continuer ? | |
| 1. Votre ami vous a parlé du jeu. Il vous a dit que ce jeu se trouve dans Concerto. Vous êtes maintenant dans Concerto. Plus précisément le jeu se trouve dans le module Accordons-nous. Votre première tâche est de trouver le jeu et l’ouvrir. Allez-y! | | b-   Quels sont les différents modules proposés dans Concerto?  b-   Avez-vous vu qu’en bas de l’écran, il y a un menu qui propose différentes activités qui peuvent être utiles? | |
| 1. Lisez d’abord les règles et commencez la partie | | b- faites attention aux boutons présents dans la première page du jeu | |
| 1. Choisissez le tas SENTIMENT ET RELATION et ouvrez-le | | b- Avez-vous repéré les 4 tas de cartes sur l’écran? | |
| 1. Tournez la première carte | | b- Est-ce qu’il y a un élément sur la carte qui permet de la tourner? | |
| 1. Défilez la carte, une fois à gauche et une fois à droite pour voir les autres présentes dans ce tas | | b- Comment faites-vous normalement dans votre smartphone pour aller à droite ou à gauche ? | |
| 1. Classez la première carte dans J’HESITE | | b- Quand on hésite on se fait des interrogations, essayez de regarder les boutons | |
| 1. Classez les trois cartes suivantes comme TRÈS IMPORTANTES | | b- Selon vous, qu’est-ce qui représentent les icônes en bas de la carte? | |
| 1. Quittez ce tas | | b- Qu'est-ce que vous voyez en haut de l’écran? | |
| 1. Ouvrez le tas QUESTIONS PRATIQUES | | b- Est-ce qu’en bas de l’écran y a-t-il des éléments utiles? | |
| 1. Classez les premières 9 cartes comme TRÈS IMPORTANTES | | b- Qu'est-ce qui représentent les différents types de coeur, selon vous? | |
| 1. [Apparition pop-up] Lisez ce message et fermez le | | b- normalement comment vous faites pour retourner en arrière?  b- qu'est-ce les premières icônes semblent représenter?  b- rappelez-vous de modifier les cartes très importantes  b- Attention, il faut modifier les cartes comme “importantes”  b- Est-ce qu’en en bas de l'écran il y a la possibilité de passer à la suite? | |
| 1. Trouvez les cartes en surplus et défilez-les. | | b- est-ce que pouvez remarquer des indices en haut qui sont en lien avec le message d’avant en pop-up?  b- comment avez-vous fait avant pour défiler les cartes? | |
| 1. Classez les cartes en surplus dans IMPORTANT et ensuite, confirmez | | b- En regardant les icônes, selon vous laquelle représente mieux le concept d’IMPORTANT? | |
| 1. Classez les cartes suivantes comme PAS IMPORTANT | | b- En regardant les icônes en bas, lesquelles semblent représenter une chose pas importante? | |
| 1. Savez-vous combien de cartes sont présentes dans chaque catégorie de classification (très important, important, pas important, j’hésite)? | | b- Avant vous avez pu modifier la classification des cartes. Est-ce que vous vous souvenez comment on y est arrivé? | |
| 1. Vous pouvez passer à l’étape suivante | | b- Est-ce que vous remarquez un bouton relatif à l’étape suivante dans l’écran? | |
| 1. Lisez les instructions et continuez le jeu | | b- comment avez-vous fait pour fermer le message avant? | |
| 1. Précisez la première carte en rajoutant du texte dans l’espace dédié (i.e. une lettre) | | b- Est-ce que vous voyez un espace pour écrire?  b- Si vous n’arrivez pas à fermer le box par un bouton, vous pouvez essayer de cliquer ailleurs | |
| 1. Validez la carte pour passer à la suivante, en sachant qu’il faut encore régler les actions | | b- Regardez après la carte s’il y a des éléments intéressants. Est-ce que quelque chose a changé? | |
| 1. Imaginez que vous avez tout à coup un doute sur les règles du jeu et aimeriez les consulter. Où trouvez-vous les règles du jeu ? | |  | |
| 1. Validez les cartes suivantes (sans les préciser) comme REGLEE et continuez la partie | | b- Regardez la première option. Est-elle similaire pour les autres cartes?  b- est-ce que vous voyez des boutons importants qui peuvent être utiles à la suite du jeu? | |
| 1. Relisez vos précisions et cochez toutes les cases | | b- Est-ce que sur la carte il y a des indices relatifs à vos précisions?  b- Est-ce que vous vous souvenez de comment on avait fait avant pour tourner la carte? | |
| 1. Rajouter du texte dans l’espace dédié (i.e. une lettre) | | b- Est-ce que vous vous souvenez comment vous avez fait pour écrire du texte? Essayez de trouver un espace pour écrire du texte | |
| 1. Validez la carte | | b- Est-ce qu’en bas de l’écran il y a des éléments qui permettent de poursuivre le jeu? | |
| 1. Exportez vos résultats et retournez au jeu | | b- Quelles sont les options proposées à la fin du texte?  b- Normalement dans votre smartphone, comment est-ce que vous retournez dans l’app ? | |
| 1. Quittez le jeu en effaçant les données | | b- Vous avez terminé le jeu. Quels sont les boutons proposés?  b- Quelle est la différence entre les boutons? | |
| **System Usability Scale questionnaire** | | | |
| N° | Question | | Options de réponse  5pt échelle de Likert (1 - fortement en désaccord; 5 – fortement d’accord) |
| 1 | Je pense que j'aimerais utiliser ce système (jeu) fréquemment. | |  |
| 1bis | Je pense que si j'ai besoin de me questionner sur ce qui est important pour ma fin de vie, j'utiliserais en priorité ce jeu. | |  |
| 2 | J'ai trouvé le système (jeu) inutilement complexe. | |  |
| 3 | J'ai trouvé le système (jeu) facile à utiliser. | |  |
| 4 | Je pense que j'aurais besoin du soutien d'une personne technique pour pouvoir utiliser ce système (jeu). | |  |
| 5 | J'ai trouvé que les différentes fonctions de ce système (jeu) étaient bien intégrées. | |  |
| 6 | J'ai trouvé qu'il y avait trop d'incohérence dans ce système (jeu). | |  |
| 7 | J'imagine que la plupart des gens apprendront à utiliser ce système (jeu) très rapidement. | |  |
| 8 | J'ai trouvé le système (jeu) très lourd à utiliser. | |  |
| 9 | Je me suis sentie très à l'aise en utilisant le système (jeu). | |  |
| 10 | J'ai dû apprendre beaucoup de choses avant de pouvoir utiliser ce système (jeu). | |  |
| **AttrakDiff questionnaire** | | | |
| Mesure | Item (proposé aux utilisateurs selon le format standard) | | Options de réponse  7pt échelle de Likert (1 - fortement d’accord avec l’adjectif à gauche; 7 – fortement d’accord avec l’adjectif à droite) |
| Échelle qualité pragmatique | Humain-Technique | |  |
|  | Simple -Compliqué | |  |
|  | Pratique-Pas pratique | |  |
|  | Fastidieux-Efficace | |  |
|  | Prévisible -Imprévisible | |  |
|  | Confus-Clair | |  |
|  | Incontrôlable-Maîtrisable | |  |
| Échelle qualité hédonique - stimulation | Original -Conventionnel | |  |
|  | Sans imagination-Créatif | |  |
|  | Prudent-Audacieux | |  |
|  | Novateur -Conservateur | |  |
|  | Ennuyeux-Captivant | |  |
|  | Peu exigeant-Challenging | |  |
|  | Nouveau-Commun | |  |
| Échelle qualité hédonique- identité | M’isole-Me sociabilise | |  |
|  | Professionnel-Amateur | |  |
|  | De bon goût-De mauvais goût | |  |
|  | Bas de gamme-Haut de gamme | |  |
|  | M’exclut-M’intègre | |  |
|  | Me rapproche des autres-M'éloigne des autres | |  |
|  | Présentable -Non présentable | |  |
| Échelle attractivité globale | Plaisant-Déplaisant | |  |
|  | Laid-Beau | |  |
|  | Agréable-Désagreable | |  |
|  | Rebutant-Attirant | |  |
|  | Bon-Mauvais | |  |
|  | Repoussant-Attrayant | |  |
|  | Motivant-Décourageant | |  |
| **MARS – Section F : Game-specific** | | | |
| Type | Question | | Option de réponse 5pt échelle de Likert (1 – Pas du tout d’accord; 2-plutôt en désaccord; 3-Indifférent; 4-plutôt d’accord; 5 – Tout à fait d’accord) |
| Sensibilisation | Ce jeu peut faire **prendre conscience de l'importance** de l'anticipation des situations difficiles qui pourraient survenir dans le futur. | |  |
| Connaissance | Ce jeu peut **permettre de** **mieux connaître/comprendre** l'importance de l'anticipation des situations difficiles qui pourraient survenir dans le futur. | |  |
| Attitudes | Ce jeu peut **changer l'attitude** face à l'anticipation des situations difficiles qui pourraient survenir dans le futur. | |  |
| Intention de changement | Ce jeu peut **augmenter l'intention et la motivatio**n à l'anticipation des situations difficiles qui pourraient survenir dans le futur. | |  |
| Recherche d’aide | Ce jeu peut **encourager à chercher plus d'aide** pour l'anticipation des situations difficiles qui pourraient survenir dans le futur. | |  |
| Changement de comportement | Ce jeu peut **améliorer** l'anticipation des situations difficiles qui pourraient survenir dans le futur. | |  |
| **MARS – Section E : Game subjective quality** | | | |
| N° | Question | | Options de réponse |
| 1 | Recommanderiez-vous ce jeu à d’autres personnes ? | | 1-Pas du tout, je ne recommanderais ce jeu à personne  2-Il y a très peu de personnes à qui je recommanderais ce jeu  3- Je recommanderais ce jeu à plusieurs personnes  4-Je recommanderais ce jeu à de nombreuses personnes  5-Je recommanderais ce jeu à tout le monde |
| **Questions de débriefing** | | | |
| **N°** | **Question** | | **Option de réponse** |
| 1 | Qu’est-ce que vous avez apprécié dans ce jeu et pourquoi? | | Texte libre |
| 2 | Qu’est-ce que vous avez moins aimé ou quelles difficultés avez-vous rencontrées avec ce jeu? | | Texte libre |
| 3 | Quelle tâche vous a semblé difficile ou compliquée? | |  |
| 4 | Est-ce que quelque chose vous a manqué dans ce jeu? | | Texte libre |
| 5 | Avez-vous encore quelque chose à ajouter? | | Texte libre |

## English Translation of Questionnaires

| **Demographic and technical questions** | | |
| --- | --- | --- |
| N° | Question | Answer’s options |
| 1 | Gender | Female; Male; Other |
| 2 | Age | Number |
|  | What is the highest level of schooling you have achieved? | Elementary school; Vocational Apprenticeship; High School (College); Federal Vocational Baccalaureate; Professional higher education ; University or EPFL |
| 3 | What type of smartphone do you currently own? | IOS; Android; Other; I don’t know; I don’t have a smartphone |
| 4 | How often do you use your smartphone? | Every day; Several times a week; Several times a month; A few times a year; Never |
| 5 | Have you ever taken a usability test? Here's how it goes... | Yes; No; Other |
| 6 | The aim is not to evaluate you, but to assess how easy the game is to use.  It's normal for some tasks to be difficult. You'll have plenty of time to do each task, and I'm here to help and guide you. Do these tasks normally, as if you were doing them for yourself.  I'm going to ask you to think aloud, to express whatever comes into your head as you carry out the various tasks.  Do you have any questions? | Free text |
| **5 seconds test** | | |
| First, an image from a stage of the game will be displayed for 5 seconds. Then, several questions will be asked to gather your impressions of this page. Please respond as honestly as possible. This will take no more than 5 minutes. When you are ready, you may express your thoughts aloud, and I will click on the "Start" button below. | | |
| N° | Question | Answer’s options |
| 1 memory dump question | What element of the game did you remember? | Free text |
| 2 Target identification questions | In your opinion, what is the goal of the presented game? | Free text |
| 3 attitudinal question | What general impression did this game give you? | Very bad; Bad; Average; Good; Very good |
| 4 attitudinal question | How do you rate the aesthetics of this game? | Very attractive; Attractive; Average; Unsightly; Very unsightly |

| **Think aloud** | | | | |
| --- | --- | --- | --- | --- |
| You are a 50-year-old man/woman who has just met your sister, who has been ill for a long time. The discussion with her leads you to think about the future, but you're not sure how to define your thoughts on advance directives. So you discuss it with a friend, who suggests you try the game *Anticip'action* located in the HUG Concerto app. | | | | |
| Task | | Inputs (a-exploration ; b-hint; ca-answer)   - 1. Yes, there are lots of things on this screen. Can you... + repeat the task.   2. I can see you're having difficulty, and that's understandable. I can give you a hint   3. It's not that simple. Try clicking here, then here. We're there! Well done! Now on to the next task.   **Reframing phrase if they digress too much:** You still have other tasks to do, and I don't want to take up too much of your time. Do you agree to continue? | | |
| 1. Your friend told you about the game. He told you that the game is in *Concerto*. You are now in *Concerto*. More precisely, the game is in the *Accordons-nous* module. Your first task is to find the game and open it. Let ’s go! | | b-   What modules does Concerto offer?  b-   Did you see that at the bottom of the screen, there's a menu offering various activities that might be useful? | | |
| 1. First, read the rules and start the game. | | b- Pay attention to the buttons on the first page of the game. | | |
| 1. Select the “Feelings and relationship” pile and open it. | | b- Can you see the 4 piles of cards on the screen? | | |
| 1. Flip the first card. | | b- Is there anything on the card that allows you to turn it? | | |
| 1. Scroll the card once to the left and once to the right to see the other cards in this pile. | | b- How do you normally go left or right on your smartphone? | | |
| 1. Classify the first card under “unsure”. | | b- When you're hesitating, try looking at the buttons. | | |
| 1. Classify the next three cards as “very important”. | | b- What do you think the icons at the bottom represent? | | |
| 1. Exit this pile. | | b- What do you see at the top of the screen? | | |
| 1. Open the “Practical questions” pile | | b- Are there any useful elements at the bottom of the screen? | | |
| 1. Classify the first nine cards as “very important” | | b- What do you think represents the different types of heart? | | |
| 1. [Pop-up appears] Read this message and close it. | | b- How do you normally go back when you use your smartphone?  b- What do the first icons seem to represent?  b- Remember to modify very important cards  b- Be careful, you have to modify the maps as “important”.  b- At the bottom of the screen, is there an option to move on? | | |
| 1. Find the surplus cards and scroll through them | | b- Are there any clues at the top that relate to the previous pop-up message?  b- How did you scroll through the cards before? | | |
| 1. Classify the surplus cards as “important” and then confirm. | | b- Looking at the icons, which do you think best represents the concept of important? | | |
| 1. Classify the following cards as “not important.” | | b- Looking at the icons at the bottom, which ones seem to represent something not important? | | |
| 1. Do you know how many cards are present in each classification category (“very important”, “important”, “not important”, “unsure”)? | | b- You were able to change the classification of the cards before. Do you remember how this was done? | | |
| 1. You can proceed to the next step. | | b- Do you notice a next-step button on the screen? | | |
| 1. [Pop-up appears] Read the instructions and continue the game. | | b- How did you close the message before? | | |
| 1. Specify the first card by adding text in the designated space (e.g., a letter). | | b- Do you see a space for writing?  b- If you can't close the box with a button, you can try clicking elsewhere. | | |
| 1. Validate the card to move on to the next one, knowing that actions still need to be resolved. | | b- Look at the card afterwards to see if there are any interesting elements. Has anything changed? | | |
| 1. Imagine that you suddenly have doubts about some game rules and would like to consult them. Where do you find the rules of the game? | |  | | |
| 1. Validate the following cards (without specifying them) as “resolved” and continue the game. | | b- Look at the first option. Is it like the other cards?  b- Do you see any important buttons that could be useful for the rest of the game? | | |
| 1. Review your specifications and check all the boxes. | | b- Are there any clues on the card relating to your specifications?  b- Do you remember how we turned the card before? | | |
| 1. Add text in the designated space (e.g., a letter). | | b- Do you remember how to write text? Try to find a space to write text | | |
| 1. Validate the card. | | b- Are there any elements at the bottom of the screen that allow you to continue the game? | | |
| 1. Export your results and return to the game. | | b- What are the options at the end of the text?  b- Normally on your smartphone, how do you return to the app? | | |
| 1. Exit the game and delete the data. | | b- You've finished the game. What buttons are available?  b- What's the difference between the buttons? | | |
| **System Usability Scale questionnaire** | | | |  |
| N° | Question  (system = game)* | | Answer’s options  5pt Likert Scale (1 – Strongly disagree; 5 – Strongly agree) |  |
| 1 | I think that I would like to use this system* frequently. | |  |  |
| 1bis | I think that if I need to question myself about what's important at the end of my life, I would give priority to this game. | |  |  |
| 2 | I found the system unnecessarily complex. | |  |  |
| 3 | I thought the system was easy to use. | |  |  |
| 4 | I think that I would need the support of a technical person to be able to use this system. | |  |  |
| 5 | I found the various functions in this system were well-integrated. | |  |  |
| 6 | I thought there was too much inconsistency in this system. | |  |  |
| 7 | I would imagine that most people would learn to use this system very quickly. | |  |  |
| 8 | I found the system very cumbersome to use. | |  |  |
| 9 | I felt confident using the system. | |  |  |
| 10 | I needed to learn a lot of things before I could get going with this system. | |  |  |
| **AttrakDiff questionnaire** | | | |  |
| Measure | Item (proposed to users in standard format) | | Options de réponse  7pt Likert Scale (1 - strongly agree with the adjective on the left; 7 - strongly agree with the adjective on the right) |  |
| Pragmatique quality | Human-Technical | |  |  |
|  | Simple-Complicated | |  |  |
|  | Practical-Impractical | |  |  |
|  | Cumbersome-Straightforward | |  |  |
|  | Predictable-Unpredictable | |  |  |
|  | Confusing-Clearly structured | |  |  |
|  | Unruly-Manageable | |  |  |
| Hedonic quality - Stimulation | Inventive-Conventional | |  |  |
|  | Unimaginative-Creative | |  |  |
|  | Bold-Cautious | |  |  |
|  | Innovative-Conservative | |  |  |
|  | Dull-Captivating | |  |  |
|  | Undemanding-Challenging | |  |  |
|  | Novel-Ordinary | |  |  |
| Hedonic quality - Identity | Isolating-Connective | |  |  |
|  | Professional-Unprofessional | |  |  |
|  | Stylish-Tacky | |  |  |
|  | Cheap-Premium | |  |  |
|  | Alienating-Integrating | |  |  |
|  | Brings me closer to people-Separates me from people | |  |  |
|  | Presentable-Unpresentable | |  |  |
| Attractiveness | Pleasant-Unpleasant | |  |  |
|  | Ugly-Attractive | |  |  |
|  | Likeable-Disagreeable | |  |  |
|  | Rejecting-Inviting | |  |  |
|  | Good-Bad | |  |  |
|  | Repelling-Appealing | |  |  |
|  | Motivating-Discouraging | |  |  |
| **MARS – Section F : Game-specific** | | | |  |
| Type | Question | | Option de réponse 5pt échelle de Likert (1 – Pas du tout d’accord; 2-plutôt en désaccord; 3-Indifférent; 4-plutôt d’accord; 5 – Tout à fait d’accord) |  |
| Awareness | This game is likely to increase awareness of the importance of addressing [the anticipation of difficult situations that may arise in the future] | |  |  |
| Knowledge | This game is likely to increase knowledge/understanding of [the anticipation of difficult situations that may arise in the future] | |  |  |
| Attitude | This game is likely to change attitudes toward improving [the anticipation of difficult situations that may arise in the future] | |  |  |
| Intention to change | This game is likely to increase intentions/motivation to address [the anticipation of difficult situations that may arise in the future] | |  |  |
| Help seeking | Use of this game is likely to encourage further help seeking [the anticipation of difficult situations that may arise in the future] | |  |  |
| Behavior change | Use of this game is likely to increase [the anticipation of difficult situations that may arise in the future] | |  |  |
| **MARS – Section E : Game subjective quality** | | | |  |
| N° | Question | | Options de réponse |  |
| 1 | Would you recommend this game to people who might benefit from it? | | 1- I would not recommend this game to anyone  2- There are very few people I would recommend this game to  3- There are several people I would recommend this game to  4- There are many people I would recommend this game to  5- I would recommend this game to everyone |  |
| **Questions de débriefing** | | | |  |
| **N°** | **Question** | | **Answer’s options** |  |
| 1 | What did you like about the game and why? | | Free text |  |
| 2 | What did you like less or what difficulties did you encounter with this game? | | Free text |  |
| 3 | What task did you find difficult or complicated? | | Free text |  |
| 4 | Did you miss anything about the game? | | Free text |  |
| 5 | Is there anything else you would like to add? | | Free text |  |
